# Robust PCA via Regularized Reaper with a Matrix-Free Proximal Algorithm

**DOI:** 10.1007/s10851-021-01019-1

**Published:** 2021-02-15

**Authors:** Robert Beinert, Gabriele Steidl

**Affiliations:** Institut für Mathematik, Technische Universität Berlin, Straße des 17. Juni 136, 10623 Berlin, Germany

**Keywords:** Robust PCA, Regularized reaper, Matrix-free PCA, PCA offset, Thick-restarted Lanczos algorithm, 58C05, 62H25, 65K10

## Abstract

Principal component analysis (PCA) is known to be sensitive to outliers, so that various robust PCA variants were proposed in the literature. A recent model, called reaper, aims to find the principal components by solving a convex optimization problem. Usually the number of principal components must be determined in advance and the minimization is performed over symmetric positive semi-definite matrices having the size of the data, although the number of principal components is substantially smaller. This prohibits its use if the dimension of the data is large which is often the case in image processing. In this paper, we propose a regularized version of reaper which enforces the sparsity of the number of principal components by penalizing the nuclear norm of the corresponding orthogonal projector. If only an upper bound on the number of principal components is available, our approach can be combined with the *L*-curve method to reconstruct the appropriate subspace. Our second contribution is a matrix-free algorithm to find a minimizer of the regularized reaper which is also suited for high-dimensional data. The algorithm couples a primal-dual minimization approach with a thick-restarted Lanczos process. This appears to be the first efficient convex variational method for robust PCA that can handle high-dimensional data. As a side result, we discuss the topic of the bias in robust PCA. Numerical examples demonstrate the performance of our algorithm.

## Introduction

Principal component analysis (PCA) [37] realizes the dimensionality reduction in data by projecting them onto those affine subspace which minimizes the sum of the squared Euclidean distances between the data points and their projections. Unfortunately, PCA is very sensitive to outliers, so that various robust approaches were developed in robust statistics [17, 28, 46] and nonlinear optimization. In this paper, we focus on the second one.

One possibility to make PCA robust consists in removing outliers before computing the principal components which has the serious drawback that outliers are difficult to identify and other data points are often falsely labeled as outliers. Another approach assigns different weights to data points based on their estimated relevance, to get a weighted PCA [20] or repeatedly estimate the model parameters from a random subset of data points until a satisfactory result indicated by the number of data points within a certain error threshold is obtained [11]. In a similar vein, least trimmed squares PCA models [38, 41] aim to exclude outliers from the squared error function, but in a deterministic way. In [44], a dual principal component pursuit is used for this purpose. The variational model in [4] decomposes the data matrix into a low rank and a sparse part. Related approaches such as [7, 32, 51] separate the low-rank component from the column sparse one using different norms in the variational model. Another group of robust PCA replaces the squared $L_2$ norm in the PCA model by the $L_1$ norm [18]. Unfortunately, this norm is not rotationally invariant, i.e., when rotating the centered data points, the minimizing subspace is not rotated in the same way. Replacing the squared Euclidean norm in the PCA model by just the Euclidean one leads to a non-convex robust PCA model with minimization over the Stiefel or Grassmannian manifold, see, e.g., [9, 26, 31, 34]. Instead of the previous model which minimizes over the sparse number of directions spanning the low-dimensional subspace, it is also possible to minimize over the orthogonal projectors onto the desired subspace. This has the advantage that the minimization can be performed over symmetric positive semi-definite matrices, e.g., using methods from semi-definite programming, and the disadvantage that the dimension of the projectors is as large as the data now. This prohibits this approach for many applications in particular in image processing. The projector PCA model is still non-convex, and a convex relaxation, called reaper, was recently proposed by Lerman et el. [27]. An extensive comparison of the benefits and drawbacks of the different approaches in the rich literature of robust PCA as well as of the related numerical algorithms can, for instance, be found in [26].

In this paper, we build up on the advantages of the convex reaper model, but modify it in two important directions: (i) by penalizing the nuclear norm of the approximated projectors, our model does only require an upper bound on the dimension of the desired subspace. Having the same effect as the sparsity promotion of the 1-norm, the nuclear norm—the 1-norm of the eigenvalues—promotes low-rank matrices or, equivalently, sparse eigenvalue decompositions; (ii) by combining primal-dual minimization techniques with a thick-restarted Lanczos process, we are able to handle high-dimensional data. We call our new convex model rreaper. We provide all computation steps leading to an efficient and a provable convergent algorithm to the minimum of the objective function. A performance analysis following the lines of [27] is given. The choice of the offset in robust PCA is an interesting problem which is not fully discussed in the literature so far. Usually, the geometric median is used. We do not provide a full solution of this issue, but show that under some assumptions the affine hyperplane in $\mathbb {R}^d$ having the smallest Euclidean distance to $n > d$ given data points goes through $d+1$ of these points. We underline our theoretical findings by numerical examples.

The outline of this paper is as follows: preliminaries from linear algebra and convex analysis are given in Sect. 2. In Sect. 3, we introduce our regularized reaper model. The basic primal-dual algorithm for its minimization is discussed in Sect. 4. The algorithm is formulated with respect to the full projection matrix. The matrix-free version of the algorithm is given in Sect. 5. It is based on the thick-restarted Lanczos algorithm and is suited for high-dimensional data. In Sect. 6, we examine the performance analysis of rreaper along the lines of [27]. Some results on the offset in robust PCA are proved in Sect. 7. The very good performance of rreaper in particular for high-dimensional data is demonstrated in Sect. 8. Further, the relation between the dimension of the wanted subspace and the regularization parameter is addressed via the *L*-curve method. Section 9 finishes the paper with conclusions and directions of future research.

## Notation and Preliminaries

Throughout this paper, we will use the following notation and basic facts from linear algebra and convex analysis which can be found in detail in various monographs and overview papers as [1, 3, 6, 13, 40].

Linear algebra By $\Vert \cdot \Vert _2$ we denote the Euclidean vector norm and by $\Vert \cdot \Vert _1$ the norm which sums up the absolute vector components. Recall that for any $\varvec{x} \in \mathbb {R}^n$,

1 $$\begin{aligned} \tfrac{1}{\sqrt{n}} \Vert \varvec{x}\Vert _1 \le \Vert \varvec{x}\Vert _2 \le \Vert \varvec{x}\Vert _1. \end{aligned}$$

Let $\mathbf {1}_n$ resp. $\mathbf {0}_n$ be the vectors having *n* entries 1, resp., 0. Analogously, we write $\varvec{1}_{n,d}$ and $\varvec{0}_{n,d}$ for the all-one and all-zero matrix in $\mathbb {R}^{n,d}$. Further, $\varvec{I}_n$ is the $n \times n$ identity matrix. Let ${\text {tr}}\varvec{A}$ denote the *trace* of the quadratic matrix $\varvec{A} \in \mathbb {R}^{n,n}$, i.e., the sum of its eigenvalues. On $\mathbb {R}^{n,d}$ the Hilbert–Schmidt inner product is defined by

$$\begin{aligned} \langle \varvec{X},\varvec{Y} \rangle :={\text {tr}}(\varvec{X}^\mathrm {T}\varvec{Y}) = {\text {tr}}(\varvec{Y} \varvec{X}^\mathrm {T}), \qquad \varvec{X},\varvec{Y} \in \mathbb {R}^{n,d}, \end{aligned}$$

and the corresponding so-called *Frobenius norm* by

$$\begin{aligned} \Vert \varvec{X}\Vert _F^2 = \langle \varvec{X} , \varvec{X} \rangle . \end{aligned}$$

Let $\mathscr {S}(n) \subset \mathbb {R}^{n,n}$ denote the linear subspace of symmetric matrices. For two symmetric matrices $\varvec{A},\varvec{B} \in \mathscr {S}(n)$, we write $\varvec{A} \preceq \varvec{B}$ if $\varvec{B} - \varvec{A}$ is positive semi-definite. Every $\varvec{A} \in \mathscr {S}(n)$ has a spectral decomposition

$$\begin{aligned} \varvec{A} = \varvec{U} {\text {diag}}(\varvec{\lambda }_{\varvec{A}}) \, \varvec{U}^\mathrm {T}, \end{aligned}$$

where $\varvec{\lambda }_{\varvec{A}} \in \mathbb {R}^n$ denotes the vector containing the eigenvalues of $\varvec{A}$ in descending order $\lambda _1 \ge \cdots \ge \lambda _n$ and $\varvec{U}$ is the orthogonal matrix having the corresponding orthogonal eigenvectors as columns. The nuclear norm (trace norm) of $\varvec{A} \in \mathscr {S}(n)$ is given by

$$\begin{aligned} \Vert \varvec{A}\Vert _{{\text {tr}}} :=\sum _{j=1}^n |\lambda _j|. \end{aligned}$$

The trace and Frobenius norm correspond to the Schatten 1-norm and 2-norm, respectively, where the Schatten *p*-norm with $1 \le p \le \infty$ of a symmetric matrix $\varvec{A}$ is defined by $||\varvec{A}||_{S_p} :=||\varvec{\lambda }_{\varvec{A}}||_p$. Recall that $\varvec{\varPi } \in \mathbb {R}^{n,n}$ is an orthogonal projector if $\varvec{\varPi } \in \mathscr {S}(n)$ and $\varvec{\varPi }^2 = \varvec{\varPi }$. This is equivalent to the statement that $\varvec{\varPi } \in \mathscr {S}(n)$ and has only eigenvalues in $\{0,1\}$. The *nuclear norm* is the unique norm such that

$$\begin{aligned} \mathrm {rank} (\varvec{\varPi }) = \Vert \varvec{\varPi }\Vert _{{\text {tr}}} \end{aligned}$$

for every orthogonal projector $\varvec{\varPi }$.

For a given norm $\Vert \cdot \Vert$ on $\mathbb {R}^n$, the *dual norm* is defined by

$$\begin{aligned} \Vert \varvec{x}\Vert _* :=\max _{\Vert \varvec{y}\Vert \le 1} \langle \varvec{x},\varvec{y} \rangle . \end{aligned}$$

In particular, for a matrix $\varvec{X} = (\varvec{x}_1|\ldots |\varvec{x}_N) \in \mathbb {R}^{n,N}$ we will be interested in the norm

$$\begin{aligned} ||\varvec{X}||_{2,1} :=\sum _{k=1}^N ||\varvec{x}_k||_2, \end{aligned}$$

which can be considered as norm on $\mathbb {R}^{nN}$ by arranging the columns of the matrix into a vector. Its dual norm is given by

$$\begin{aligned} ||\varvec{X}||_{2,1,*} = ||\varvec{X}||_{2,\infty } :=\max _{k=1,\ldots ,N} ||\varvec{x}_k||_2. \end{aligned}$$

Convex analysis Let $\varGamma _0(\mathbb {R}^n)$ denote the space of proper, lower semi-continuous, convex functions mapping from $\mathbb {R}^n$ into the extended real numbers $(-\infty ,\infty ]$. The *indicator function* $\iota _{\mathscr {C}}$ of $\mathscr {C} \subseteq \mathbb {R}^n$ is defined by

$$\begin{aligned} \iota _{\mathscr {C}}(\varvec{x}) = \left\{ \begin{array}{ll} 0 &{}\quad \mathrm {if} \; \varvec{x} \in \mathscr {C},\\ + \infty &{}\quad \mathrm {otherwise}. \end{array} \right. \end{aligned}$$

We have $\iota _{\mathscr {C}} \in \varGamma _0(\mathbb {R}^n)$ if and only if $\mathscr {C}$ is non-empty, convex and closed.

For $f \in \varGamma _0(\mathbb {R}^n)$, the *proximal mapping* is defined by

$$\begin{aligned} {\text {prox}}_{f}(\varvec{x}) :=\mathop {{\text {argmin}}}\limits _{\varvec{y} \in \mathbb {R}^n} \left\{ f(\varvec{y}) + \tfrac{1}{2} ||\varvec{x} - \varvec{y}||_2^2 \right\} . \end{aligned}$$

Indeed, the minimizer exists and is unique [40, Thm. 31.5]. If $\mathscr {C} \subset \mathbb {R}^n$ is a non-empty, closed, convex set, then the proximal mapping of a multiple of $\iota _{\mathscr {C}}$ is just the orthogonal projection onto $\mathscr {C}$, i.e.,

$$\begin{aligned} {\text {prox}}_{\sigma \iota _{\mathscr {C}}}(\varvec{x}) = {\text {proj}}_{\mathscr {C}} (\varvec{x}), \quad \sigma >0. \end{aligned}$$

In particular, the orthogonal projection onto the halfspace $\mathscr {H}(\varvec{a},\beta ) :=\{\varvec{x} \in \mathbb {R}^n: \langle \varvec{a} , \varvec{x} \rangle \le \beta \}$ with $\varvec{a} \in \mathbb {R}^{n}$ and $\beta \in \mathbb {R}$ can be computed by

$$\begin{aligned} {\text {proj}}_{\mathscr {H}(\varvec{a},\beta )} (\varvec{x}) = \varvec{x} - \frac{(\langle \varvec{a}, \varvec{x} \rangle - \beta )_+}{\Vert \varvec{a}\Vert _2^2} \, \varvec{a}, \end{aligned}$$

where $(y )_+ :=\max \{0,y\}$. Further, the orthogonal projection onto the hypercube $\mathscr {Q} := [0,1]^n$ is given by

2 $$\begin{aligned} {\text {proj}}_{\mathscr {Q}} (\varvec{x}) = \left( \max \left\{ \min \{ x_j,1 \},0 \right\} \right) _{j=1}^n. \end{aligned}$$

The *Fenchel dual* of $f \in \varGamma _0(\mathbb {R}^n)$ is the function $f^* \in \varGamma _0(\mathbb {R}^n)$ defined by

$$\begin{aligned} f^*(\varvec{p}) :=\max _{\varvec{x} \in \mathbb {R}^n}\langle \varvec{p},\varvec{x} \rangle - f(\varvec{x}). \end{aligned}$$

The dual function of a norm is just the indicator function of the unit ball with respect to its dual norm. In particular, we have for $\Vert \cdot \Vert _{2,1}: \mathbb {R}^{n,N} \rightarrow \mathbb {R}$ that

3 $$\begin{aligned} \Vert \varvec{X} \Vert _{2,1}^* = \iota _{\mathscr {B}_{2,\infty }}(\varvec{X}), \end{aligned}$$

where $$ \mathscr {B}_{2,\infty } :=\{\varvec{X} \in \mathbb {R}^{n,N}: \Vert \varvec{x}_k \Vert _2 \le 1 \; \mathrm {for \; all} k=1,\ldots ,N\} $$.

## Regularized reaper

Given *N* data points $\varvec{x}_1,\ldots ,\varvec{x}_N \in \mathbb {R}^n$, the classical PCA finds a *d*-dimensional affine subspace $\{\varvec{A} \, \varvec{t} + \varvec{b}: \varvec{t} \in \mathbb {R}^d\}$, $1 \le d \ll n$, by minimizing

4 $$\begin{aligned} \begin{aligned} \sum _{k=1}^N \min _{t \in \mathbb {R}^d} \Vert \varvec{A}\, \varvec{t} + \varvec{b} - \varvec{x}_k \Vert _2^2&= \sum _{k=1}^N \Vert (\varvec{A} \varvec{A}^\mathrm {T}- \varvec{I}_n )(\varvec{b}-\varvec{x}_k)\Vert _2^2\\&\quad \mathrm {subject } \;\mathrm{to} \quad \varvec{A}^\mathrm {T}\varvec{A} = \varvec{I}_d \end{aligned} \end{aligned}$$

over $\varvec{b} \in \mathbb {R}^n$ and $\varvec{A} \in \mathbb {R}^{n,d}$. It is not hard to check that the affine subspace goes through the *offset* (bias)

5 $$\begin{aligned} \bar{\varvec{b}} :=\tfrac{1}{N}(\varvec{x}_1 + \ldots + \varvec{x}_N). \end{aligned}$$

Therefore, we can reduce our attention to data points $\varvec{x}_k - \bar{\varvec{b}}$, $k=1,\ldots ,N$, which we denote by $\varvec{x}_k$ again, and minimize over the linear *d*-dimensional subspaces through the origin, i.e.,

$$\begin{aligned} \min _{\varvec{A} \in \mathbb {R}^{n,d}} \sum _{k=1}^N \Vert (\varvec{A} \varvec{A}^\mathrm {T}- \varvec{I}_n ) \varvec{x}_k \Vert _2^2 \quad \mathrm {subject } \; \mathrm{to} \quad \varvec{A}^\mathrm {T}\varvec{A} = \varvec{I}_d, \end{aligned}$$

where $$\varvec{X} :=(\varvec{x}_1| \ldots |\varvec{x}_N) \in \mathbb {R}^{n,N}$$.

Unfortunately, the solution of this minimization problem is sensitive to outliers. Therefore, several robust PCA variants were proposed in the literature. A straightforward approach consists in just skipping the square in the Euclidean norm leading to

6 $$\begin{aligned} \begin{aligned}&\min _{\varvec{A} \in \mathbb {R}^{n,d}} \sum _{k=1}^N \Vert (\varvec{A} \varvec{A}^\mathrm {T}- \varvec{I}_n ) \varvec{x}_k \Vert _2 = \Vert \varvec{A} \varvec{A}^\mathrm {T}\varvec{X} - \varvec{X}\Vert _{2,1} \\&\quad \mathrm {subject }\;\mathrm{to} \quad \varvec{A}^\mathrm {T}\varvec{A} = \varvec{I}_d. \end{aligned} \end{aligned}$$

This is a non-convex model which requires the minimization over matrices $\varvec{A}$ in the so-called Stiefel manifold,

$$\begin{aligned} \mathrm {St}(n,d) :=\{\varvec{A} \in \mathbb {R}^{n,d}: \varvec{A}^\mathrm {T}\varvec{A} = \varvec{I}_d \}, \end{aligned}$$

see [9, 26, 34, 35].

Another approach is based on the observation that $\varvec{\varPi } :=\varvec{A} \varvec{A}^\mathrm {T}$ is the orthogonal projector onto the linear subspace spanned by the columns of $\varvec{A}$. Since the linear subspace is *d*-dimensional, exactly *d* eigenvalues of $\varvec{\varPi }$ have to be one. Thus, problem (6) can be reformulated as

7 $$\begin{aligned} \begin{aligned}&\min _{\varPi \in \mathscr {S}(n) } \Vert \varvec{\varPi } \varvec{X} - \varvec{X} \Vert _{2,1}\\&\quad \mathrm {subject } \; \mathrm{to} \quad \varvec{\lambda }_{\varvec{\varPi }} \in \{0,1\}^n, \; {\text {tr}}(\varvec{\varPi }) = d. \end{aligned} \end{aligned}$$

Having computed $\varvec{\varPi }$, we can determine $\varvec{A}$ by spectral decomposition. Unfortunately, (7) is still a non-convex model which is moreover NP hard to solve. Therefore, Lerman et al. [27] suggested to replace it by a convex relaxation, called reaper,

$$\begin{aligned} \min _{P \in \mathscr {S}(n) } \Vert \varvec{P} \varvec{X} \!-\! \varvec{X} \Vert _{2,1} \quad \mathrm {subject } \; \mathrm{to} \quad \varvec{0}_{n,n} \!\preceq \! \varvec{P} \!\preceq \! \varvec{I}_n, \; {\text {tr}}(\varvec{P}) \!=\! d. \end{aligned}$$

In order to deal with the non-differentiability of the objective function, Lerman et al. [27] iteratively solve a series of positive semi-definite programs. In contrast to models minimizing directly over $\varvec{A} \in \mathbb {R}^{n,d}$, algorithms for minimizing reaper or rreaper seem to require the handling of a large matrix $\varvec{P} \in \mathscr {S}(n)$ or, more precisely, the handling of its spectral decomposition which makes the method not practicable for high-dimensional data.

The above model requires the exact knowledge of the dimension *d* of the linear subspace the data will be reduced to. In this paper, we suggest to replace the strict trace constraint by a relaxed variant ${\text {tr}}(\varvec{\varPi }) \le d$ and to add the nuclear norm of $\varvec{\varPi }$ as a regularizer which enforces the sparsity of the rank of $\varvec{\varPi }$:

8 $$\begin{aligned} \begin{aligned}&\min _{\varPi \in \mathscr {S}(n) } \Vert \varvec{\varPi } \varvec{X} - \varvec{X} \Vert _{2,1} + \alpha \Vert \varvec{\varPi }\Vert _{{\text {tr}}}\\&\quad \mathrm {subject } \; \mathrm{to} \quad \varvec{\lambda }_{\varvec{\varPi }} \in \{0,1\}^n, \; {\text {tr}}(\varvec{\varPi }) \le d. \end{aligned} \end{aligned}$$

Here $\alpha >0$ is an appropriately fixed regularization parameter.

Since (8) is again hard so solve, we use a relaxation for the eigenvalues and call the new model regularized reaper (rreaper):

9 $$\begin{aligned} \begin{aligned}&\min _{P \in \mathscr {S}(n) } \Vert \varvec{P} \varvec{X} - \varvec{X} \Vert _{2,1} + \alpha \Vert \varvec{P}\Vert _{{\text {tr}}}\\&\quad \mathrm {subject } \; \mathrm{to} \quad \varvec{0}_{n,n} \preceq \varvec{P} \preceq \varvec{I}_n, \; {\text {tr}}(\varvec{P}) \le d. \end{aligned} \end{aligned}$$

Finally, we project the solution of rreaper to the set of orthoprojectors with rank not larger than *d*:

$$\begin{aligned} \mathscr {O}_d :=\{ \varvec{\varPi } \in \mathscr {S}(n): \varvec{\lambda }_{\varvec{\varPi }} \in \mathscr {E}_d\}, \end{aligned}$$

where

$$\begin{aligned} \mathscr {E}_d :=\{ \lambda \in \mathbb {R}^n: \varvec{\lambda } \in \{0,1\}^n, \, \langle \varvec{\lambda }, \varvec{1}_n \rangle \le d\}. \end{aligned}$$

In the following, we will present a primal-dual approach to solve (9) which uses only the sparse spectral decomposition of $\varvec{P}$, but not the matrix itself within the computation steps.

## Primal-Dual Algorithm

Rreaper is a convex optimization problem; so we may choose from various convex solvers. Before choosing a specific method, we study the structure of rreaper in more details. For this purpose, we define the forward operator

$$\begin{aligned} \mathscr {X} :\mathscr {S}(n) \rightarrow \mathbb {R}^{n, N} : \varvec{P} \mapsto \varvec{P} \varvec{X} \end{aligned}$$

and rearrange (9) as

10 $$\begin{aligned} \min _{\varvec{P} \in \mathscr {S}(n)} ||\mathscr {X} (\varvec{P}) - \varvec{X}||_{2,1} + \alpha \mathscr {R} (\varvec{P}) , \end{aligned}$$

where the regularizer $\mathscr {R} :\mathscr {S}(n) \rightarrow [0,+\infty ]$ is defined by

11 $$\begin{aligned} \begin{aligned}&\mathscr {R}(\varvec{P}) :=||\varvec{P}||_{{\text {tr}}} + \iota _{\mathscr {C}}(\varvec{P}), \\&\mathscr {C} :=\{ \varvec{P} \in \mathscr {S}(n) : \varvec{0}_{n,n} \preceq \varvec{P} \preceq \varvec{I}_n, {\text {tr}}( \varvec{P}) \le d \}. \end{aligned} \end{aligned}$$

Since $\mathscr {C}$ is compact and convex, and since the norms $||\cdot ||_{2,1}$ and $||\cdot ||_{{\text {tr}}}$ are continuous, rreaper has a global minimizer. This minimizer is in general not unique. Concerning the adjoint operator $\mathscr {X}^*: \mathbb {R}^{n, N} \rightarrow \mathscr {S}(n)$, we observe

$$\begin{aligned} \langle \mathscr {X}(\varvec{P}),\varvec{Y}\rangle&= \tfrac{1}{2} \bigl ( \langle \varvec{P} \varvec{X},\varvec{Y}\rangle + \langle \varvec{P}^\mathrm {T}\varvec{X},\varvec{Y}\rangle \bigr )\\&= \tfrac{1}{2} \bigl ( {\text {tr}}( \varvec{Y}^\mathrm {T}\varvec{P} \varvec{X}) + {\text {tr}}(\varvec{X}^\mathrm {T}\varvec{P} \varvec{Y}) \bigr )\\&= \tfrac{1}{2} \left\langle \varvec{P},\tfrac{1}{2}(\varvec{X} \varvec{Y}^\mathrm {T}+ \varvec{Y} \varvec{X}^\mathrm {T}) \right\rangle . \end{aligned}$$

for all $\varvec{P} \in \mathscr {S}(n)$ and $\varvec{Y} \in \mathbb {R}^{n,N}$, where we exploit the symmetry of $\varvec{P}$ by $\varvec{P} = \nicefrac {1}{2}(\varvec{P} + \varvec{P}^\mathrm {T})$. Thus, the adjoint is just

$$\begin{aligned} \mathscr {X}^*(\varvec{Y}) = \tfrac{1}{2}(\varvec{X} \varvec{Y}^\mathrm {T}+ \varvec{Y} \varvec{X}^\mathrm {T}). \end{aligned}$$

The operator norm of $\mathscr {X}$ is given by the spectral norm of $\varvec{X} \in \mathbb {R}^{n,N}$, i.e.,

$$\begin{aligned} \Vert \mathscr {X}\Vert = \Vert \varvec{X}\Vert _2. \end{aligned}$$

In more detail, for $\varvec{P} = (\varvec{p}_1|\ldots |\varvec{p}_n) \in \mathscr {S}(n)$, we obtain

$$\begin{aligned} \Vert \mathscr {X}\Vert&= {\mathop {\mathop {\max }\limits _{\varvec{P} \in \mathscr {S}(n)}}\limits _{\Vert \varvec{P}\Vert _F \le 1}} \Vert \varvec{P} \varvec{X}\Vert _F = {\mathop {\mathop {\max }\limits _{\varvec{P} \in \mathscr {S}(n)}}\limits _{\Vert \varvec{P}\Vert _F \le 1}} \biggl (\sum _{j=k}^n \Vert \varvec{X}^\mathrm {T}\varvec{p}_k\Vert _2^2\biggr )^\frac{1}{2} \\&\le {\mathop {\mathop {\max }\limits _{\varvec{P} \in \mathscr {S}(n)}}\limits _{\Vert \varvec{P}\Vert _F \le 1}} \biggl (\Vert \varvec{X}\Vert _2^2 \sum _{k=1}^n \Vert \varvec{p}_k\Vert _2^2\biggr )^\frac{1}{2} \le \Vert \varvec{X}\Vert _2. \end{aligned}$$

Here the inequality becomes sharp for

$$\begin{aligned} \varvec{P} = \varvec{U} {\text {diag}}( (1,0,\ldots ,0)^\mathrm {T}) \, \varvec{U}^\mathrm {T}, \end{aligned}$$

where $\varvec{U}$ arises from the singular value decomposition $\varvec{X} = \varvec{U} {\text {diag}}( \varvec{\sigma }_{\varvec{X}}) \, \varvec{V}^\mathrm {T}$ with descending ordered singular values $\sigma _1 \ge \cdots \ge \sigma _{\min \{n,N\}}$.

More generally, rreaper is an optimization problem of the form

12 $$\begin{aligned} {\text {minimize}}\quad F(\mathscr {A}(\varvec{P})) + G(\varvec{P}), \end{aligned}$$

whose objective consists of two convex, lower semi-continuous functions $F = ||\cdot - \varvec{X}||_{2,1}$ and $G = \alpha \mathscr {R}$ and a linear mapping $\mathscr {A} = \mathscr {X}$. Since both—data fidelity and regularizer of rreaper—are non-differentiable, gradient decent methods as well as the forward-backward splitting or the fast iterative shrinkage-thresholding algorithm (FISTA) cannot be applied. In order to exploit the structure, various saddle-point methods that do not require differentiability can be used. Examples are the alternating directions method of multipliers (ADMM) which is related to the Douglas–Rachford splitting or primal-dual algorithms, see for instance [6] and references therein. Since the iteration variables with respect to $\varvec{P}$ in rreaper computed via ADMM usually have no sparse spectral decomposition and cannot be handled for high-dimensional instances, we prefer to choose the primal-dual method of Chambolle and Pock [6] with extrapolation of the primal variable to compute the minimizer of rreaper (10).

For the general minimization problem (12), the primal-dual method consists in the iteration

$$\begin{aligned} \varvec{Y}^{(r+1)}&:={\text {prox}}_{\sigma F^*} \bigl ( \varvec{Y}^{(r)} + \sigma \, \mathscr {A} (\bar{\varvec{P}}^{(r)}) \bigr )\\ \varvec{P}^{(r+1)}&:={\text {prox}}_{\tau G} \bigl ( \varvec{P}^{(r)} - \tau \mathscr {A}^*(\varvec{Y}^{(r+1)} ) \bigr )\\ \bar{\varvec{P}}^{(r+1)}&:=(1 + \theta ) \, \varvec{P}^{(r+1)} - \theta \, \varvec{P}^{(r)} \end{aligned}$$

with fixed parameters $\tau , \sigma >0$ and $\theta \in [0,1]$. The extrapolation step consisting in the calculation of $\bar{\varvec{P}}^{(r+1)}$ is here required to ensure the convergence whenever $\tau \sigma < 1 / ||\mathscr {A}||^2$ and $\theta = 1$, where $||\mathscr {A}||$ denotes the operator norm of $\mathscr {A}$. If the functions *F* and *G* possesses more regularity like strong convexity, the parameter $\theta$ may be varied to prove an acceleration of the convergence and to guarantee certain convergence rates [5, 6]. For rreaper, the above iteration leads us to the following numerical method.

### Algorithm 1

**(Primal-Dual Algorithm)** Input: $\varvec{X} \in \mathbb {R}^{n,N}$ $d \in \mathbb {N}$, and $\sigma ,\tau > 0$ with $\sigma \tau < 1/\Vert \varvec{X}\Vert _2^2$, and $\theta \in (0,1]$.

Intialization: $${\varvec{P}}^{(0)} = \bar{\varvec{P}}^{(0)} = \varvec{0}_{n,n}$$ , $$\varvec{Y}^{(0)} :=\varvec{0}_{n, N}$$.

Iteration:

$$\begin{aligned} \varvec{Y}^{(r+1)}&:={\text {prox}}_{\sigma ||\cdot \, - \varvec{X}||_{2,1}^*} \Bigl ( \varvec{Y}^{(r)} + \sigma \mathscr {X} \bigl (\bar{\varvec{P}}^{(r)} \bigr ) \Bigr ),\\ \varvec{P}^{(r+1)}&:={\text {prox}}_{\tau \alpha \mathscr {R}} \Bigl ( \varvec{P}^{(r)} - \tau \mathscr {X}^* \bigl (\varvec{Y}^{(r+1)} \bigr ) \Bigr ),\\ \bar{\varvec{P}}^{(r+1)}&:=(1 + \theta ) \, \varvec{P}^{(r+1)} - \theta \, \varvec{P}^{(r)} \end{aligned}$$

More generally, Chambolle and Pock [6] have proven that the sequence $\{\varvec{P}^{(r)} \}_{r\in \mathbb {N}}$ converges to a minimizer $\hat{\varvec{P}}$ of (10) and the sequence $\{\varvec{Y}^{(r)} \}_{r\in \mathbb {N}}$ to a minimizer of the dual problem

$$\begin{aligned} \min _{\varvec{Y} \in \mathbb {R}^{n,N}} \Vert \cdot - X\Vert _{2,1}^*(\varvec{Y}) + (\alpha \mathscr {R})^*(- \mathscr {X}^*(\varvec{Y}) ) \end{aligned}$$

if the Lagrangian

13 $$\begin{aligned} L(\varvec{P},\varvec{Y}) :=- \Vert \cdot - X\Vert _{2,1}^*(\varvec{Y}) + \alpha \mathscr {R}(\varvec{P}) + \langle \mathscr {X}(\varvec{P}), \varvec{Y} \rangle \end{aligned}$$

has a saddle-point which is, however, clear for rreaper.

The algorithm requires the computation of the proximal mapping of the dual data fidelity and of the regularizer which we consider next.

### Proposition 1

**(Proximal mapping of the dual data fidelity)** For $\varvec{x} \in \mathbb {R}^{n,N}$ and $\sigma > 0$, we have

$$\begin{aligned} {\text {prox}}_{\sigma ||\cdot \, - \varvec{X}||_{2,1}^*} = {\text {proj}}_{\mathfrak {B}_{2,\infty }} ( \cdot - \sigma \varvec{X}). \end{aligned}$$

### Proof

Using (3) and, since $(f(\cdot - x_0))^* = f^* + \langle \cdot , x_0 \rangle$, we obtain

$$\begin{aligned}&{\text {prox}}_{\sigma ||\cdot \, - \varvec{X}||_{2,1}^*} (\varvec{Y})\\&\quad = \mathop {{\text {argmin}}}\limits _{\varvec{Z} \in \mathbb {R}^{n,N}} \bigl \{\tfrac{1}{2} \Vert \varvec{Z}-\varvec{Y}\Vert _F^2 + \iota _{\mathfrak {B}_{2,\infty }}(\varvec{Z}) + \sigma \langle \varvec{Z},\varvec{X} \rangle \bigr \}\\&\quad = \mathop {{\text {argmin}}}\limits _{\varvec{Z} \in \mathbb {R}^{n,N}} \bigl \{ \tfrac{1}{2} \Vert \varvec{Z}-(\varvec{Y}-\sigma \varvec{X})\Vert _F^2 + \iota _{\mathfrak {B}_{2,\infty }}(\varvec{Z})\bigr \}\\&\quad = {\text {proj}}_{\mathfrak {B}_{2,\infty }} ( Y - \sigma \varvec{X}). \end{aligned}$$

For the maximal dimension *d* of the target subspace, we henceforth use the half-space

$$\begin{aligned} \mathscr {H} :=\mathscr {H}(\varvec{1}_n,d) = \{\varvec{x} \in \mathbb {R}^n: \langle \varvec{x},\varvec{1}_n \rangle \le d \}. \end{aligned}$$

in order to bound the trace of the primal iteration variable $\varvec{P}^{(r)}$. Then the proximal mapping of the regularizer is given in the following proposition.

### Proposition 2

**(Proximal mapping of the regularizer)** For $\varvec{P} \in \mathscr {S}(n)$ with spectral decomposition $\varvec{P} = \varvec{U} {\text {diag}}(\varvec{\lambda }_{\varvec{P}}) \, \varvec{U}^\mathrm {T}$ and $\mathscr {R}$ in (11) it holds

$$\begin{aligned} {\text {prox}}_{\tau \alpha \mathscr {R}}(\varvec{P}) = \varvec{U} {\text {diag}}({\text {proj}}_{\mathscr {Q}\cap \mathscr {H}}(\varvec{\lambda }_{\varvec{P}} - \tau \alpha \varvec{1}_n)) \, \varvec{U}^\mathrm {T}. \end{aligned}$$

### Proof

A symmetric matrix $\varvec{P}$ is in $\mathscr {C}$ if and only if $\varvec{\lambda }_{\varvec{P}}$ $\in$ $\mathscr {Q} \cap \mathscr {H}$. Hence, the regularizer can be written as

$$\begin{aligned} \mathscr {R} (\varvec{P}) = \langle \varvec{\lambda }_{\varvec{P}}, \varvec{1}_n \rangle + \iota _{\mathscr {Q} \cap {\mathscr {H}}}(\varvec{\lambda }_{\varvec{P}}). \end{aligned}$$

and

14 $$\begin{aligned} \begin{aligned}&{\text {prox}}_{\tau \alpha \mathscr {R}}(\varvec{P})\\&\quad = \mathop {{\text {argmin}}}\limits _{\varvec{S} \in \mathscr {S}(n)} \bigl \{ \tfrac{1}{2} \Vert \varvec{S} - \varvec{P}\Vert _F^2 \\&\qquad + \tau \alpha \langle \varvec{\lambda }_{\varvec{S}}, \varvec{1}_n \rangle + \iota _{\mathscr {Q} \cap {\mathscr {H}}}(\varvec{\lambda }_{\varvec{S}}) \bigr \}. \end{aligned} \end{aligned}$$

By the theorem of Hoffmann and Wielandt [16, Thm. 6.3.5], we know that

$$\begin{aligned} \Vert \varvec{S} - \varvec{P}\Vert _F^2 \ge \Vert \varvec{\lambda }_{\varvec{S}} - \varvec{\lambda }_{\varvec{P}} \Vert _2^2 \end{aligned}$$

with equality if and only if $\varvec{S}$ possesses the same eigenspaces as $\varvec{P}$. Therefore, the minimizer in (14) has to be of the form $\varvec{S} = \varvec{U} {\text {diag}}(\varvec{\lambda }_{\varvec{S}}) \, \varvec{U}^\mathrm {T}$, where the columns of $\varvec{U}$ are the eigenvectors of $\varvec{P}$. Incorporating this observation in (14), we determine the eigenvalues $\varvec{\lambda }_{\varvec{S}}$ by solving the minimization problem

$$\begin{aligned} \varvec{\lambda }_{\varvec{S}}&= \mathop {{\text {argmin}}}\limits _{\varvec{\lambda }_{\varvec{S}} \in \mathbb {R}^n} \Bigl \{ \tfrac{1}{2} \Vert \varvec{\lambda }_{\varvec{S}} - \varvec{\lambda }_{\varvec{P}}\Vert _2^2 \\&\quad + \tau \alpha \langle \varvec{\lambda }_{\varvec{S}}, \varvec{1}_n \rangle + \iota _{\mathscr {Q} \cap {\mathscr {H}}}(\varvec{\lambda }_{\varvec{S}}) \Bigr \}\\&= \mathop {{\text {argmin}}}\limits _{\varvec{\lambda }_{\varvec{S}}} \Bigl \{ \tfrac{1}{2} \Vert \varvec{\lambda }_{\varvec{S}} + \tau \alpha \varvec{1}_n - \varvec{\lambda }_{\varvec{P}}\Vert _2^2 + \iota _{\mathscr {Q} \cap {\mathscr {H}}}(\varvec{\lambda }_{\varvec{S}}) \Bigr \}\\&= {\text {proj}}_{\mathscr {Q}\cap {\mathscr {H}}}(\varvec{\lambda }_{\varvec{P}} - \tau \alpha \varvec{1}_n)). \end{aligned}$$

$$\square $$

Alternatively to the proof, we could argue with the so-called spectral function related to $\mathscr {R}$ which is invariant under permutations, see, e.g., [1].

By Proposition 2, the proximal mapping of the regularizer requires the projection onto the truncated hypercube. The following proposition can be found in [1, Ex. 6.32].

### Proposition 3

**(Projection onto the truncated hypercube)** For any $\varvec{\lambda } \in \mathbb {R}^n$ and any $d \in (0,n]$, the projection to the truncated hypercube is given by

$$\begin{aligned} {\text {proj}}_{\mathscr {Q}\cap {\mathscr {H}}}(\varvec{\lambda }) = \left\{ \begin{array}{ll} {\text {proj}}_{\mathscr {Q} } (\varvec{\lambda }) &{}\quad \mathrm {if} \; \langle {\text {proj}}_{\mathscr {Q} } (\varvec{\lambda }), \varvec{1}_n \rangle \le d,\\ {\text {proj}}_{\mathscr {Q} } (\varvec{\lambda } - \hat{t} \varvec{1}_n)&{}\quad \mathrm {otherwise} , \end{array} \right. \end{aligned}$$

where $\hat{t}$ is the smallest root of the function

15 $$\begin{aligned} \varphi (t) :=\langle {\text {proj}}_{\mathscr {Q} }( \varvec{\lambda } - t \varvec{1}_n ), \varvec{1}_n \rangle - d, \quad t \ge 0. \end{aligned}$$

Due to the projection to the hypercube, see (2), only the positive components of $\varvec{\lambda }$ influence its projection onto $\mathscr {Q}\cap {\mathscr {H}}$. More precisely, we have

$$\begin{aligned} {\text {proj}}_{\mathscr {Q}\cap {\mathscr {H}}}(\varvec{\lambda }) = {\text {proj}}_{\mathscr {Q}\cap {\mathscr {H}}}(\varvec{\lambda })_+ , \end{aligned}$$

where the function $(\cdot )_+$ is employed componentwise.

To formulate a projection algorithm, in particular, to compute the zero of $\varphi$, we study the properties of $\varphi$ and derive an explicit representation. Within a different setting, the reader may also have a look at [30].

### Lemma 1

**(Properties of** $\varvec{\varphi }$) For fixed $\varvec{\lambda } \in \mathbb {R}^n$ with $\langle {\text {proj}}_{\mathscr {Q}}(\varvec{\lambda }),\varvec{1}_n\rangle > d$, the function $\varphi :[0,\infty ) \rightarrow \mathbb {R}$ defined in (15) has the following properties:

(i) $\varphi$ is Lipschitz continuous.
(ii) $\varphi$ is monotone decreasing and piecewise linear. More precisely, we can construct a sequence $0 = s_0< s_1< s_2< \ldots < s_M$ with $M \le 2n$ such that
  $$\begin{aligned} \varphi (t) = \varphi (s_l) - k_l (t-s_l), \end{aligned}$$
  for $$t \in [s_l,s_{l+1})$$, $$l = 0,\ldots , M-1$$, where
  $$\begin{aligned} k_l :=|\{j \in \{1,\ldots ,n\}: (\varvec{\lambda } - s_l \varvec{1}_n)_j \in (0,1] \}|. \end{aligned}$$
  Moreover, $$\varphi (t) = -d$$ for $$t \ge s_M$$.
(iii) The smallest root $\hat{t}$ of $\varphi$ is given by
  $$\begin{aligned} \hat{t} = s_m + \tfrac{1}{k_m} \varphi (s_m) , \end{aligned}$$
  where *m* is the unique index such that $\varphi (s_m) > 0$ and $\varphi (s_{m+1}) \le 0$.

### Proof

(i) Using the definition of $\varphi$, the Cauchy–Schwarz inequality, and the non-expansiveness of the projection, we get
  $$\begin{aligned}&|\varphi (t) - \varphi (s)|\\&\quad = |\langle {\text {proj}}_{\mathscr {Q} }(\varvec{\lambda } - t \varvec{1}_n ), \varvec{1}_n \rangle - \langle {\text {proj}}_{\mathscr {Q} }(\varvec{\lambda } - s\varvec{1}_n ), \varvec{1}_n \rangle |\\&\quad \le \sqrt{n} \, \Vert {\text {proj}}_{\mathscr {Q} }(\varvec{\lambda } - t \varvec{1}_n ) -{\text {proj}}_{\mathscr {Q} }(\varvec{\lambda } - s\varvec{1}_n )\Vert _2\\&\quad \le \sqrt{n} \, \Vert (s-t) \varvec{1}_n\Vert _2 = n \, |s-t|. \end{aligned}$$
(ii) Starting with $s_0 = 0$, we construct $s_l$ with $l=1,\ldots ,M$ iteratively as follows: given $s_l$ , we set $\varvec{\mu } :=\varvec{\lambda } - s_l \varvec{1}_n$ and choose
  $$\begin{aligned} s_{l+1} :=s_{l} + h_l, \quad h_l := \min \{s_{\mathrm {leave}},s_{\mathrm {enter}} \}, \end{aligned}$$
  where
  $$\begin{aligned} s_{\mathrm {leave}}&:=\min _j \{\mu _j: \mu _j \in (0,1]\},\\ s_{\mathrm {enter}}&:=\min _j \{\mu _j-1: \mu _j >1\}. \end{aligned}$$
  Here we use the convention $\min \emptyset = \infty$. If both sets in the definition of $s_{\mathrm {leave}}$ and $s_{\mathrm {enter}}$ are empty, we stop the construction since all components of $\varvec{\mu }$ are non-positive implying ${\text {proj}}_{\mathscr {Q} }(\varvec{\mu }) = \varvec{0}_n$ and thus $\varphi (s_M) = -d$ as well as $\varphi (t) = -d$ for $t \ge s_M$, where *M* is the index of the last computed point $s_l$. Considering the projection to the hypercube $\mathscr {Q}$ in (2), we see that the index set $\{j \in \{1,\ldots ,n\}: (\varvec{\lambda } - t \varvec{1}_n)_j \in (0,1] \}$ does not change for $t \in [s_l, s_{l+1})$ and that a change appears exactly in $s_{l+1}$, where at least one component enters or leaves the interval (0, 1]. Hence, we have
  $$\begin{aligned} \varphi (t) = \varphi (s_l) - k_l (t-s_l), \quad t \in [s_l,s_{l+1}). \end{aligned}$$
  Let $s_{M}$ be the first value in this procedure, where all components of $\varvec{\mu }$ become non-positive. Since each component in $\varvec{\lambda } - t \varvec{1}_n$ can at most one times enter or leave the interval (0, 1], we know that $M < 2n$. Further, $k_l \ge 0$ shows that $\varphi$ is monotone decreasing.
(iii) By definition of $\varphi$ and by the assumption $\langle {\text {proj}}_{\mathscr {Q}}(\varvec{\lambda }),\varvec{1}_n\rangle > d$, we have $\varphi (0) > 0$. On the other side, $\varphi (s_m) = -d$ was shown above. Consequently, the smallest zero $\hat{t}$ of $\varphi$ has to lie in the interval $[s_{m},s_{m+1}]$ with $\varphi (s_m) > 0$ and $\varphi (s_{m+1}) \le 0$ and can be computed by solving
  $$\begin{aligned} \varphi (\hat{t}) = \varphi ( s_m) - k_m (\hat{t}-s_m) = 0. \end{aligned}$$
  This results in $\hat{t} = s_m + \tfrac{1}{k_m} \varphi (s_m)$ and finishes the proof. $\square$

Following Proposition 3 and the previous proof, we obtain the following algorithm for the projection onto $\mathscr {Q} \cap \mathscr {H}$.

### Algorithm 2

**(Projection onto truncated hypercube)**Input: $\varvec{\lambda } \in \mathbb {R}^n$, $d \in \mathbb {N}$.

(i) Compute $$\varvec{\mu } :={\text {proj}}_{\mathscr {Q}} (\varvec{\lambda })$$ by (2).
  If $\langle \varvec{\mu }, \varvec{1}_n \rangle \le d$, then return $\hat{\varvec{\lambda }} = \varvec{\mu }$;
  otherwise set $s :=0$, $\varphi :=+ \infty$ and $\varvec{\mu } = \varvec{\lambda }$.
(ii) Repeat until $\varphi \le 0$:
  - $$s_{\mathrm {old}} :=s$$,
  - $$s_{\mathrm {leave}} :=\min _j \{\mu _j: \mu _j \in (0,1]\}$$,
  - $$s_{\mathrm {enter}} :=\min _j \{\mu _j-1: \mu _j >1\}$$,
  - $$s :=s + \min \{s_{\mathrm {leave}},s_{\mathrm {enter}}\}$$,
  - $$\varvec{\mu } = \varvec{\lambda } - s \varvec{1}_n$$,
  - $$\varphi = \langle {\text {proj}}_{\mathscr {Q}} (\varvec{\mu }), \varvec{1}_n \rangle - d$$,
(iii) Compute
  - $$k :=|\{j \in \{1,\ldots ,n\}: (\varvec{\lambda } - s_{\mathrm {old}} \varvec{1}_n)_j \in (0,1] \}|$$,
  - $$ \hat{t} = s_{\mathrm {old}} + \tfrac{1}{k} \varphi (s_{\mathrm {old}}). $$

Output: $$\hat{\varvec{\lambda }} :={\text {proj}}_{\mathscr {Q}\cap {\mathscr {H}}}(\varvec{\lambda })$$.

Based on the derived proximal mappings, the primal-dual Algorithm 2 to solve rreaper (10) can be specified in matrix form as follows.

### Algorithm 3

**(Primal-dual** **r** **reaper**) Input: $\varvec{X} \in \mathbb {R}^{n, N}$, $d \in \mathbb {N}$, $\alpha > 0$, and $\sigma ,\tau > 0$ with $\sigma \tau < 1/\Vert \varvec{X}\Vert _2^2$, and $\theta \in [0,1)$.

Initiation: $$\varvec{P}^{(0)} = \bar{\varvec{P}}^{(0)}:=\varvec{0}_{n, n}$$, $$\varvec{Y}^{(0)} :=\varvec{0}_{n, N}$$.

Iteration:

(i) Dual update:
  $$\begin{aligned} \varvec{Y}^{(r+1)} :={\text {proj}}_{\mathfrak {B}_{2, \infty }} \bigl ( \varvec{Y}^{(r)} + \sigma \bigl (\mathscr {X} \bigl (\bar{\varvec{P}}^{(r)} \bigr ) - \varvec{X} \bigr ) \bigr ). \end{aligned}$$
(ii) Primal update:
  - $$\varvec{U} {\text {diag}}(\varvec{\lambda }) \, \varvec{U}^\mathrm {T}:=\varvec{P}^{(r)} - \tau \mathscr {X}^* \bigl (\varvec{Y}^{(r+1)} \bigr )$$,
  - $$\hat{\varvec{\lambda }} :={\text {proj}}_{_{\mathscr {Q}\cap {\mathscr {H}}}}(\varvec{\lambda } - \tau \alpha \varvec{1}_n)$$    (Algorithm 2),
  - $$\varvec{P}^{(r+1)} :=\varvec{U} {\text {diag}}(\hat{\varvec{\lambda }}) \, \varvec{U}^*$$.
(iii) Extrapolation: $$ \bar{\varvec{P}}^{(r+1)} :=(1 + \theta ) \, \varvec{P}^{(r+1)} - \theta \, \varvec{P}^{(r)}$$.

Output: $\hat{\varvec{P}}$ (Solution of rreaper (10)).

## Matrix-Free Realization

Solving rreaper with the primal-dual Algorithm 3 is possible if the dimension of the surrounding space $\mathbb {R}^n$ is moderate which is often not the case in image processing tasks. While the dual variable $\varvec{Y} \in \mathbb {R}^{n,N}$ matches the dimension of the data, the primal variable $\varvec{P}$ is in *S*(*n*) instead of $\mathbb {R}^{n,d}$, $d \ll n$. How can the primal-dual iteration be realized in the case $n \gg d$ though the primal variable cannot be hold in memory and the required eigenvalue decomposition cannot be computed in a reasonable amount of time?

Here the nuclear norm in rreaper that promotes low-rank matrices comes to our aid. Our main idea to derive a practical implementation of the primal-dual iteration is thus based on the assumption that the iterates of the primal variable $\varvec{P}^{(r)}$ possess the form

16 $$\begin{aligned} \varvec{P}^{(r)} :=\sum _{k=1}^{d_r} \lambda _k^{(r)} \, \varvec{u}_k^{(r)} \, \bigl (\varvec{u}_k^{(r)}\bigr )^\mathrm {T}\end{aligned}$$

with small rank $d_r$. In our simulations, we observed that the rank is usually around the dimension *d* of the wanted low-dimensional subspace.

In order to integrate the matrix-free representation (16) into the primal-dual iteration efficiently, we further require a fast method to compute the eigenvalue thresholding. For this, we compute a partial eigenvalue decomposition using the well-known Lanczos process [22]. Deriving matrix-free versions of the forward operator $\mathscr {X}$ and its adjoint $\mathscr {X}^*$, we finally introduce a complete matrix-free primal-dual implementation with respect to $\varvec{P}^{(r)}$.

### The Thick-Restarted Lanczos Process

One of the most commonly used methods to extract a small set of eigenvalues and their corresponding eigenvectors of a large symmetric matrix is the Lanczos method [22], which is based on theory of Krylov subspaces. The method builds a partial orthogonal basis first and then uses a Rayleigh–Ritz projection to extract the wanted eigenpairs approximately. If the set of employed basis vectors is increased, the extracted eigenpairs converge to the eigenpairs of the given matrix [13]. Since the symmetric matrix whose partial eigenvalue decomposition is required in the primal-dual method usually is high-dimensional, we would like to chose the number $k_{\mathrm {max}}$ of basis vectors within the Lanczos method as small as possible such that the convergence of the Krylov method does not emerge. To calculate the dominant $\ell _{\mathrm {fix}}$ eigenpairs with high-accuracy nevertheless, the Lanczos method can be restarted with the dominant $\ell _{\mathrm {fix}}$ Ritz pairs. For our purpose, we use the thick-restart scheme of Wu and Simon [50] in Algorithm 4, whose details are discussed below.

#### Algorithm 4

**(Thick-restarted Lanczos process** [50, Alg. 3]**)**Input: $\varvec{P} \in S(n)$, $k_{\mathrm {max}}> \ell _{\mathrm {fix}} > 0$, $\delta > 0$.

(i) Choose a unit vector $\varvec{r}_0 \in \mathbb {R}^n$. Set $\ell :=0$.
(ii) Lanczos process:
  1. Initiation:
    - $$\varvec{e}_{\ell + 1} :=\varvec{r}_\ell / ||\varvec{r}_\ell ||_2$$,
    - $$\varvec{q} :=\varvec{P} \varvec{e}_{\ell + 1}$$,
    - $$\beta _{\ell + 1} :=\langle \varvec{q},\varvec{e}_{\ell + 1}\rangle $$,
    - $$\varvec{r}_{\ell + 1} :=\varvec{q} - \beta _{\ell + 1} \varvec{e}_{\ell + 1} - \sum _{k=1}^\ell \rho _k \varvec{e}_k$$,
    - $$\gamma _{\ell + 1} :=||\varvec{r}_{\ell + 1}||$$.
  2. Iteration ($$k = \ell + 2, \dots , k_{\mathrm {max}}$$):
    - $$\varvec{e}_k :=\varvec{r}_{k-1} / \gamma _{k-1}$$,
    - $$\varvec{q} :=\varvec{P} \varvec{e}_k$$,
    - $$\beta _k :=\langle \varvec{q},\varvec{e}_k\rangle $$,
    - $$\varvec{r}_k :=\varvec{q} - \beta _k \varvec{e}_k - \gamma _{k-1} \varvec{e}_{k-1}$$,
    - $$\gamma _k :=||\varvec{r}_k||$$.
(iii) Compute the eigenvalue decomposition $\varvec{T} = \varvec{Y} \varvec{\varLambda }\varvec{Y}^\mathrm {T}$ of $\varvec{T}$ in (17). Set $\varvec{U} :=\varvec{E} \varvec{Y}$.
(iv) If $\gamma _{k_{\mathrm {max}}} |y_{k_{\mathrm {max}},k}| \le \delta ||\varvec{P}||$ for $k = 1, \dots , \ell _{\mathrm {fix}}$, then return $\varvec{U} :=[\varvec{u}_1 | \dots | \varvec{u}_{\ell _{\mathrm {fix}}}]$ and $\varvec{\varLambda }:={\text {diag}}(\lambda _1, \dots , \lambda _{\ell _{\mathrm {fix}}})$. Otherwise, set $\ell :=\ell _{\mathrm {fix}}$, $\varvec{r}_\ell :=\varvec{r}_{k_{\mathrm {max}}}$, and continue with (ii).

Output: $$\varvec{U} \in \mathbb {R}^{n \times \ell _{\mathrm {fix}}}$$, $$\varvec{\varLambda }\in \mathbb {R}^{\ell _{\mathrm {fix}} \times \ell _{\mathrm {fix}}}$$ with $$\varvec{U}^\mathrm {T}\varvec{P} \varvec{U} = \varvec{\varLambda }$$.

#### Remark 1

Although the Lanczos process computes an orthogonal basis $\varvec{e}_1, \dots , \varvec{e}_{k_{\mathrm {max}}}$, the orthogonality is usually lost because of the floating-point arithmetic. In order to re-establish the orthogonality, we therefore have to orthogonalize the newly computed $\varvec{e}_k$ with the previous basis vectors, which can be achieved by the Gram–Schmidt procedure. More sophisticated re-orthogonalization strategies are discussed in [50].

#### Remark 2

During the Lanczos process, the norm of the residual $\gamma _k$ could become zero. In this case, we can stop the process, reduce $k_{\mathrm {max}}$ to the current *k*, and proceed with step (iii) and (iv). Then the computed basis $\varvec{e}_1, \dots , \varvec{e}_k$ spans an invariant subspace of $\varvec{P}$ such that the eigenpairs in $\varvec{U}$ and $\varvec{\varLambda }$ become exact, see [13].

The heart of the Lanczos method in Algorithm 4 is the construction of an orthogonal matrix $\varvec{E} :=[\varvec{e}_1 | \dots | \varvec{e}_{k_{\mathrm {max}}}] \in \mathbb {R}^{n \times k_{\mathrm {max}}}$ such that $\varvec{T} :=\varvec{E}^\mathrm {T}\varvec{P} \varvec{E}$ becomes tridiagonal, see (17) with $\ell = 0$ below. Using the eigenvalue decomposition $\varvec{T} = \varvec{Y} \varvec{\varLambda }\varvec{Y}^\mathrm {T}$, we then compute the Ritz pairs $(\lambda _k, \varvec{u}_k)$, where $\varvec{u}_k$ are the columns of $\varvec{U} :=[\varvec{u}_1 | \dots | \varvec{u}_{k_{\mathrm {max}}}]$ and $\lambda _k$ the eigenvalues in $\varvec{\varLambda }$. In the next iteration, we chose $\ell _{\mathrm {fix}}$ Ritz pairs corresponding to the absolute leading Ritz values denoted by $(\breve{\lambda }_1, \breve{\varvec{u}}_1), \dots , (\breve{\lambda }_{\ell _{\mathrm {fix}}}, \breve{\varvec{u}}_{\ell _{\mathrm {fix}}})$ and restart the Lanczos process. Thereby, the chosen Ritz vectors are extended to an orthogonal basis $\varvec{E} :=[\breve{\varvec{u}}_1 | \dots | \breve{\varvec{u}}_{\ell _{\mathrm {fix}}} | \varvec{e}_{\ell _{\mathrm {fix}}+1} | \dots | \varvec{e}_{k_{\mathrm {max}}}]$ fulfilling

17 $$\begin{aligned} \varvec{E}^* \varvec{P} \varvec{E} = \varvec{T} = \begin{bmatrix} \breve{\lambda }_1 &{} &{} &{} \rho _1 \\ &{} \ddots &{} &{} \vdots \\ &{} &{} \breve{\lambda }_\ell &{} \rho _\ell \\ \rho _1 &{} \cdots &{} \rho _\ell &{} \beta _{\ell +1} &{} \gamma _{\ell +1} \\ &{} &{} &{} \gamma _{\ell +1} &{} \beta _{\ell +1} &{} \ddots \\ &{} &{} &{} &{} \ddots &{} \ddots &{} \gamma _{k_{\mathrm {max}}-1} \\ &{} &{} &{} &{} &{} \gamma _{k_{\mathrm {max}}-1} &{} \beta _{k_{\mathrm {max}}} \\ \end{bmatrix}, \end{aligned}$$

where $\rho _k :=\breve{\gamma }_{k_{\mathrm {max}}} \breve{y}_{k_{\mathrm {max}}, k}$ with $\breve{\gamma }_{k_{\mathrm {max}}}$ and $\breve{y}_{k_{\mathrm {max}}, k}$ originating from the last iteration, see [50].

The stopping criteria of the thick-restarted Lanczos process is here deduced from the fact that the chosen Ritz pairs fulfill the equation

$$\begin{aligned} \varvec{P} \, \breve{\varvec{u}}_k = \breve{\lambda }_k \, \breve{\varvec{u}}_k + \breve{y}_{k_{\mathrm {max}}, k} \, \breve{\varvec{r}}_{k_{\mathrm {max}}}, \end{aligned}$$

where $\breve{\varvec{r}}_{k_{\mathrm {max}}}$ is the last residuum vector of the previous iteration [50]. Consequently, the absolute error of the chosen Ritz pairs is given by

$$\begin{aligned} ||\varvec{P} \, \breve{\varvec{u}}_k - \breve{\lambda }_k \, \breve{\varvec{u}}_k||_2 = |\breve{y}_{k_{\mathrm {max}}, k}| \, ||\breve{\varvec{r}}_{k_{\mathrm {max}}}||_2 =\breve{\gamma }_{k_{\mathrm {max}}} \, |\breve{y}_{k_{\mathrm {max}}, k}|. \end{aligned}$$

Usually, the absolute value of the leading Ritz value is a good approximation of the required spectral norm $||\varvec{P}||$ to estimate the current relative error.

#### Remark 3

(*Power method*) Besides the Krylov subspace methods like the discussed Lanczos process with thick-restarting, the required eigenpairs may be calculated using the power method. Here the main idea is to start with an orthogonal matrix $\varvec{U}^{(0)} :=[\varvec{u}_1^{(0)} | \dots | \varvec{u}_{\ell _\mathrm {fix}}^{(0)}] \in \mathbb {R}^{n \times \ell _{\mathrm {fix}}}$ representing an $\ell _{\mathrm {fix}}$-dimensional subspace $\mathscr {U}^{(0)}$ and to successively compute $\mathscr {U}^{(r)} :=\varvec{P} ( \mathscr {U}^{(r-1)})$ again represented by an orthogonal matrix $\varvec{U}^{(r)}$. The convergence of the so-called orthogonal iteration may be improved by constructing $\varvec{U}^{(r)}$ using the eigenvalue decomposition of $\varvec{P}$ restricted to $\mathscr {U}^{(r)}$. This approach goes back to [43] and consists in the iteration

18 $$\begin{aligned}&\varvec{E}^{(r)} \varvec{R}^{(r)} = \varvec{P} \varvec{U}^{(r-1)}, \end{aligned}$$

19 $$\begin{aligned}&\varvec{E}^{(r) \mathrm {T}} \varvec{P} \varvec{E}^{(r)} = \varvec{Y}^{(r)} \varvec{\varLambda }^{(r)} \varvec{Y}^{(r) \mathrm {T}} \end{aligned}$$

20 $$\begin{aligned}&\varvec{U}^{(r)} :=\varvec{E}^{(r)} \varvec{Y}^{(r)}, \end{aligned}$$

where the first step consists in the QR decomposition of $\varvec{P} \varvec{U}^{(r-1)}$ into an orthogonal matrix $\varvec{E}^{(r)}$ and an upper triangular matrix $\varvec{R}^{(r)}$ and the second in the eigenvalue decomposition of $\varvec{E}^{(r) \mathrm {T}} \varvec{P} \varvec{E}^{(r)}$.

If none of the columns of $\varvec{U}^{(0)}$ is orthogonal to the $\ell _{\mathrm {fix}}$ leading eigenvectors of $\varvec{P}$, and if the $\ell _{\mathrm {fix}}$leading eigenvalues are well-defined—both in absolute value, then the columns of $\varvec{U}^{(r)}$ and the eigenvalues in $\varvec{\varLambda }^{(r)}$ converge to the leading eigenpairs of $\varvec{P}$, see [13, 43].

Although there is no convergence guarantee for the restarted Lanczos process, it usually enjoys much faster convergence than the orthogonal iteration (18)–(20) for high-dimensional problems. Moreover, the chosen restarting technique can be easily adapted to compute the proximal mapping of the regularizer as discussed in the next section.

### Matrix-Free Primal Update

The thick-restarted Lanczos method allows us to compute the leading absolute eigenvalues and their corresponding eigenvectors in a matrix-free manner using only the action of the considered matrix. In our primal-dual method for rreaper, we need the action of $\varvec{P}^{(r)} - \tau \mathscr {X}^*(\varvec{Y}^{(r+1)})$. Incorporating the low-rank representation (16), we see that this can be rewritten as

$$\begin{aligned} \varvec{e} \in \mathbb {R}^n&\mapsto \left\{ \sum _{k=1}^{d_r} \lambda _k^{(r)} \, \bigl \langle \varvec{e},\varvec{u}_k^{(r)}\bigr \rangle \, \varvec{u}_k^{(r)} \right\} \\&\quad - \frac{\tau }{2} \, \left\{ \varvec{Y}^{(r+1)} \, \bigl [\varvec{X}^\mathrm {T}\varvec{e} \bigr ] + \varvec{X} \, \bigl [\bigl ( \varvec{Y}^{(r+1)} \bigr )^\mathrm {T}\varvec{e}\bigr ] \right\} . \end{aligned}$$

For the evaluation of the primal proximal mapping, we first compute the eigenvalue decomposition of $\varvec{P}^{(r)} - \tau \mathscr {X}^*(\varvec{Y}^{(r+1)})$, next shift the eigenvalues, and finally project them to the truncated hypercube $\mathscr {Q} \cap \mathscr {H}$, see Algorithm 3. Since the projection onto $\mathscr {Q} \cap \mathscr {H}$ is independent of negative eigenvalues, see note after Proposition 3, it is thus sufficient to compute only the eigenpairs with eigenvalue larger than $\alpha \tau$.

For the numerical implementation, we compute the relevant eigenpairs with the thick-restarted Lanczos method. In the course of this, we are confronted with the issue that we actually do not know how many eigenpairs has to be computed. To reduce the overhead of Algorithm 4 as much as possible, the parameters $\ell _{\mathrm {fix}}$ and $k_{\mathrm {max}}$ can be easily adapted between the restarts. Further, the computation of strongly negative eigenvalues can be avoided by an eigenvalue shift, i.e., actually compute the eigenpairs of $\varvec{P}^{(r)} - \tau \mathscr {X}^*(\varvec{Y}^{(r+1)}) + \nu \varvec{I}$ with $\mu \ge 0$, where the required action has the form

21 $$\begin{aligned} \begin{aligned} \varvec{e} \in \mathbb {R}^n&\mapsto \biggl \{ \sum _{k=1}^{d_r} \lambda _k^{(r)} \, \bigl \langle \varvec{e},\varvec{u}_k^{(r)}\bigr \rangle \, \varvec{u}_k^{(r)} \biggr \}\\&\qquad - \frac{\tau }{2} \, \biggl \{ \varvec{Y}^{(r+1)} \, \bigl [\varvec{X}^\mathrm {T}\varvec{e} \bigr ] + \varvec{X} \, \bigl [\bigl ( \varvec{Y}^{(r+1)} \bigr )^\mathrm {T}\varvec{e}\bigr ] \biggr \} + \nu \,\varvec{e}. \end{aligned} \end{aligned}$$

Essentially, we may thus implement the primal proximation in the following manner.

#### Algorithm 5

**(Matrix-free primal proximation)**Input: $\varvec{P}^{(r)} \in \mathscr {S}(n)$, $\varvec{Y}^{(r+1)} \in \mathbb {R}^{n, N}$, $d > 0$, $\tau > 0$, $\alpha > 0$.

(i) Thick-restarted Lanczos method:
  Setting $\nu :=0$, $\ell _{\mathrm {fix}} :={\text {rank}}(\varvec{P}^{(r)})$, $k_{\mathrm {max}} :=\min \{2 \ell _{\mathrm {fix}}, n\}$, run Algorithm 4 with action (21). Between restarts, check convergence and update parameters:
  - If $\gamma _{k_{\mathrm {max}}} |y_{k_{\mathrm {max}},k}| \le \delta \, ||\varvec{P}^{(r)} - \tau \mathscr {X}^*(\varvec{Y}^{(r+1)}) + \nu \varvec{I}||$ for $k = 1, \dots , m+1$, and if $\lambda _1 \ge \dots \ge \lambda _m \ge \alpha \tau + \nu > \lambda _{m+1}$, then return $\varvec{U} :=[\varvec{u}_1 | \dots | \varvec{u}_m]$ and $\varvec{\varLambda }:={\text {diag}}(\lambda _1 - \nu , \dots , \lambda _m - \nu )$.
  - If $\lambda _{\ell _{\mathrm {fix}}} > \alpha \tau + \nu$, then increase $\ell _{\mathrm {fix}}$, $k_{\mathrm {max}}$ so that $\ell _{\mathrm {fix}} < k_{\mathrm {max}} \le n$.
  - Set $$\xi :=\max \{[\lambda _1]_-, \dots , [\lambda _{k_{\mathrm {max}}}]_-\}$$ and $$\nu :=\nu + \xi $$.
    Restart with $$(\lambda _k + \xi , \varvec{u}_k)$$, $$k=1, \dots , \ell _{\mathrm {fix}}$$.
(ii) Projection onto $\mathscr {Q} \cap \mathscr {H}$: Run Algorithm 2 on
  $$\begin{aligned} \varvec{\lambda } :=(\lambda _1 - \alpha \tau , \dots , \lambda _m - \alpha \tau , 0, \dots , 0)^\mathrm {T}\in \mathbb {R}^n \end{aligned}$$
  to get $$\hat{\varvec{\lambda }} :={\text {proj}}_{\mathscr {Q} \cap \mathscr {H}}(\varvec{\lambda })$$.
(iii) New low-rank representation:
  Determine $d_{r+1} :=\max \{k : \hat{\lambda }_k > 0\}$ and return
  $$\begin{aligned} \varvec{P}^{(r+1)} :=\sum _{k=1}^{d_{r+1}} \hat{\lambda }_k \, \varvec{u}_k \varvec{u}_k^\mathrm {T}. \end{aligned}$$

Output: $$\varvec{P}^{(r+1)} :=\sum _{k=1}^{d_{r+1}} \lambda ^{(r+1)}_k \, \varvec{u}_k^{(r+1)} \bigl ( \varvec{u}_k^{(r+1)} \bigr )^\mathrm {T}$$.

#### Remark 4

If the matrix $\varvec{P}^{(r)} - \tau \mathscr {X}^*(\varvec{Y}^{(r+1)})$ does not possess any eigenvalues greater than $\alpha \tau$, then the Lanczos process stops in step (i.a) with $m=0$. Since the projection to the truncated hypercube is then the zero vector again, the new iteration $\varvec{P}^{(r+1)}$ can be represented by an empty low-rank representation, i.e., $d_{r+1}=0$.

### Matrix-Free Dual Update

Compared with the primal update, the derivation of the matrix-free dual update is more straightforward. First, the matrix

$$\begin{aligned} \varvec{Z} :=\varvec{Y}^{(r)} + \sigma \bigl [ \mathscr {X} \bigl ((1+\theta ) \, \varvec{P}^{(r)} - \theta \, \varvec{P}^{(r-1)}\bigr ) - \varvec{X} \bigr ] \end{aligned}$$

is computed, where the over-relaxation $\bar{\varvec{P}}^{(r)} :=(1+\theta ) \, \varvec{P}^{(r)} - \theta \, \varvec{P}^{(r-1)}$ is already plugged in. The low-rank representations of $\varvec{P}^{(r)}$ and $\varvec{P}^{(r-1)}$ similar to (16) can efficiently incorporated by calculating the matrix $\varvec{Z} :=[\varvec{z}_1 | \dots | \varvec{z}_N]$ column by column. This way of handling the forward operator $\mathscr {X}$ nicely matches with the projection of the columns $\varvec{z}_k$ to the Euclidean unit ball in the second step. Writing the matrix $\varvec{Y}^{(r)} :=[\varvec{y}_1^{(r)}| \dots | \varvec{y}_N^{(r)}]$ column by column too, we obtain the following numerical method.

#### Algorithm 6

**(Matrix-free dual proximation)**Input: $\varvec{Y}^{(r)} \in \mathbb {R}^{n,N}$, $\varvec{P}^{(r)} \in S(n)$, $\varvec{P}^{(r-1)} \in S(n)$, $\sigma > 0$,$\theta \in (0,1]$.

(i) For $$k = 1, \dots , N$$, compute
  $$\begin{aligned} \varvec{z}_k&:=\varvec{y}_k^{(r)} + \sigma \, (1 + \theta ) \, \biggl \{ \sum _{\ell =1}^{d_{r}} \lambda _\ell ^{(r)} \bigl \langle \varvec{x}_k,\varvec{u}_\ell ^{(r)}\bigr \rangle \varvec{u}_\ell ^{(r)} \biggr \} \\&\qquad - \sigma \theta \, \biggl \{ \sum _{\ell =1}^{d_{r-1}} \lambda _\ell ^{(r-1)} \bigl \langle \varvec{x}_k,\varvec{u}_\ell ^{(r-1)}\bigr \rangle \varvec{u}_\ell ^{(r-1)} \biggr \} - \sigma \varvec{x}_k. \end{aligned}$$
(ii) For $k = 1, \dots , N$, compute $\varvec{z}_k :=\varvec{z}_k / (1 + [||\varvec{z}_k||_2 - 1]_+)$.
(iii) Return $$\varvec{Y}^{(r+1)} :=[\varvec{z}_1 | \dots | \varvec{z}_N]$$.

Output: $$\varvec{Y}^{(r+1)}$$

### Matrix-Free Projection onto the Orthoprojectors

With the matrix-free implementations of the primal and dual proximal mappings, we are already able to solve rreaper (9) numerically. Before summarizing the compound algorithm, we briefly discuss the last needed component to tackle the robust PCA problem (8). The final step is to project the solution $\hat{\varvec{P}}$ of rreaper onto the set of orthoprojectors with rank not larger than *d*:

$$\begin{aligned} \mathscr {O}_d :=\{ \varvec{\varPi } \in \mathscr {S}(n): \varvec{\lambda }_{\varvec{\varPi }} \in \mathscr {E}_d\}, \end{aligned}$$

where

$$\begin{aligned} \mathscr {E}_d :=\{ \lambda \in \mathbb {R}^n: \varvec{\lambda } \in \{0,1\}^n, \, \langle \varvec{\lambda }, \varvec{1}_n \rangle \le d\}. \end{aligned}$$

We may calculate the projection explicitly in the following manner.

#### Proposition 4

**(Projection onto the orthoprojectors)** For $\varvec{P} \in S(n)$ with eigenvalue decomposition $\varvec{P} = \varvec{U} {\text {diag}}(\varvec{\lambda }_{\varvec{P}}) \varvec{U}^\mathrm {T}$, and for every $1 \le p \le \infty$, the projection onto $\mathscr {O}_d$ with respect to the Schatten *p*-norm is given by

$$\begin{aligned} {\text {proj}}_{\mathscr {O}_d}(\varvec{P}) = \varvec{U} {\text {diag}}({\text {proj}}_{\mathscr {E}_d}(\varvec{\lambda })) \, \varvec{U}^\mathrm {T}. \end{aligned}$$

#### Proof

The key ingredient to prove this statement is the theorem of Lidskii–Mirsky–Wielandt, see for instance [29]. Using this theorem to estimate the Schatten *p*-Norm, we obtain

22 $$\begin{aligned} \min _{\varPi \in \mathscr {O}_d} \Vert \varvec{P} - \varvec{\varPi } \Vert _{S_p} \ge \min _{\varvec{\lambda }_{\varvec{\varPi } }\in \mathscr {E}_d} \Vert \varvec{\lambda }_{\varvec{P} } - \varvec{\lambda }_{\varvec{\varPi } } \Vert _p, \end{aligned}$$

where we have equality if $\varvec{\varPi }$ has the same eigenvectors as $\varvec{P}$. Recall that the eigenvalues in $\varvec{\lambda }_{\varvec{P} }$ appear in descending order. The right-hand side of (22) thus becomes minimal if we choose the eigenvalues of $\varvec{\varPi }$ for $k=1,\dots , d$ as

$$\begin{aligned} \hat{\lambda }_{\varvec{\varPi },k}&:=\left\{ \begin{aligned} 1&\quad \text {if } \lambda _{\varvec{P},k} \ge \tfrac{1}{2}, \\ 0&\quad \text {if } \lambda _{\varvec{P},k} < \tfrac{1}{2}, \\ \end{aligned} \right. \end{aligned}$$

and set $\hat{\lambda }_{\varvec{\varPi },k} :=0$ for $k=d+1,\ldots ,n$. This is exactly the projection onto $\mathscr {E}_d$. $\square$

Because of the low-rank representation

$$\begin{aligned} \varvec{P}^{(r)} = \sum _{k=1}^{d_r} \lambda _k \, \varvec{u}_k^{(r)} (\varvec{u}_k^{(r)})^\mathrm {T}\end{aligned}$$

of the primal variable, the construction of the orthoprojector $\hat{\varvec{\varPi }} \in \mathscr {O}_d$ is here especially simple.

#### Algorithm 7

**(Matrix-free projection onto orthoprojectors)**Input: $\varvec{P} = \sum _{k=1}^\kappa \lambda _k \, \varvec{u}_k \varvec{u}_k^\mathrm {T}\in \mathscr {S}(n)$, $d \in \mathbb {N}$.

(i) Projection onto $$\mathscr {O}_d$$:
  Determine $$s :=\max \{ k : \lambda _k \ge \nicefrac 12, k \le \min \{d, \kappa \}\}$$.
(ii) Matrix-free presentation:
  Return $$\varvec{\varPi } = \sum _{k=1}^s \varvec{u}_k \varvec{u}_k^\mathrm {T}$$.

Output: $$\varvec{\varPi } = {\text {proj}}_{\mathscr {O}_d}(\varvec{P})$$.

### Matrix-Free Robust PCA by Rreaper

Combining the matrix-free implementations of the primal and dual proximal mappings, we finally obtain a primal-dual method to solve rreaper (9) without evaluating the primal variable $\varvec{P}^{(r)}$ representing the relaxed orthoprojector explicitly.

#### Algorithm 8

**(Matrix-free robust PCA)**Input: $\varvec{X} \in \mathbb {R}^{n, N}$, $d \in \mathbb {N}$, $\alpha > 0$, and $\sigma ,\tau > 0$ with $\sigma \tau < 1/\Vert \varvec{X}\Vert _2^2$, and $\theta \in (0,1]$.

Initiation: $$\varvec{P}^{(0)} = \bar{\varvec{P}}^{(0)}:=\varvec{0} \in \mathbb {R}^{n, n}$$, $$\varvec{Y}^{(0)} :=\varvec{0} \in \mathbb {R}^{n, N}$$.

Iteration:

(i) Dual update: Compute $\varvec{Y}^{(r+1)}$ with Algorithm 6.
(ii) Primal update: Compute $\varvec{P}^{(r+1)}$ with Algorithm 5.

Projection: Compute $\hat{\varvec{\varPi }}$ with Algorithm 7.

Output: $\hat{\varvec{\varPi }}$ (Orthoprojector onto recovered subspace).

Due to the convexity of rreaper, every minimizer of (9) is global. Thus, our proposed algorithm cannot get stuck in local minima. Since the Lagrangian (13) has a saddle-point by [40, Cor. 28.2.2], the convergence of the underlying primal-dual method to a saddle-point $(\hat{\varvec{P}}, \hat{\varvec{Y}})$ is guaranteed for parameter choices $\sigma \tau < 1/\left| \left| \varvec{X}\right| \right| _2^2$ and $\theta = 1$ as proven in [5, Thm. 1]. Finally, the primal variable $\hat{\varvec{P}}$ minimizes rreaper by [40, Thm. 28.3].

However, using the thick-restarted Lanczos process to evaluate the proximal mapping of the regularizer in Proposition 2, we introduce a systematic error since the required eigenvalue decompositions are only computed approximately. Fortunately, we may control this error by choosing the accuracy $\delta$ in Algorithm 4 appropriately small. If the accuracies becomes higher during the primal-dual iteration, we will see that the underlying primal-dual iteration converges nevertheless.

#### Theorem 1

**(Convergence of Algorithm **8**)** Let

$$\begin{aligned} \varvec{Q}^{(r)} :=\varvec{P}^{(r-1)} - \tau \mathscr {X}^*(\varvec{Y}^{(r)}) = \varvec{U} {\text {diag}}(\varvec{\lambda })\, \varvec{U}^\mathrm {T}\end{aligned}$$

be the argument in the *r*th primal-dual iteration of Algorithm 8, and $\tilde{\varvec{Q}}^{(r)}$ the computed low-rank approximation of the leading positive eigenvalues greater than $\tau \alpha$ by the thick-restarted Lanczos process. Define the approximation error by

$$\begin{aligned} E_r :=||(\tilde{\varvec{Q}}^{(r)} - \tau \alpha \varvec{I}_n)_{\succeq \varvec{0}} - (\varvec{Q}^{(r)}- \tau \alpha \varvec{I}_n)_{\succeq \varvec{0}}||_F, \end{aligned}$$

where $( \cdot )_{\succeq \varvec{0}}$ denotes the projection to the positive-definite cone. Then, for $\sigma \tau < 1 / ||\varvec{X}||_2^2$ and $\theta = 1$, the primal-dual iteration in Algorithm 8 converges to a global minimizer of rreaper whenever $\sum _{r=0}^\infty E_r^{\nicefrac 12} (||\varvec{Q}^{(r)}||_F + \sqrt{d})^{\nicefrac 12} < \infty$.

#### Proof

The key idea of the proof is to show that the approximations $\tilde{\varvec{P}}^{(r)} = {\text {prox}}_{\tau \alpha \mathscr {R}} (\tilde{\varvec{Q}}^{(r)})$ are so-called type-one approximations of the proximal points $\varvec{P}^{(r)}$ in the second step of Algorithm 1. For this, we have to compare the objective

$$\begin{aligned} F(\varvec{P}) :=\tau \alpha \, ||\varvec{P}||_{{\text {tr}}} + \iota _{\mathscr {C}}(\varvec{P}) + \tfrac{1}{2} \, ||\varvec{Q}^{(r)} - \varvec{P}||_F^2 \end{aligned}$$

of ${\text {prox}}_{\tau \alpha \mathscr {R}}$ at the points $\tilde{\varvec{P}}^{(r)}$ and $\varvec{P}^{(r)}$. By the triangle inequality, we obtain

$$\begin{aligned}&F(\tilde{\varvec{P}}^{(r)}) - F(\varvec{P}^{(r)})\\&\quad = \tau \alpha \, ||\tilde{\varvec{P}}^{(r)}||_{{\text {tr}}} + \tfrac{1}{2} \, ||\varvec{Q}^{(r)} - \tilde{\varvec{P}}^{(r)}||_F^2\\&\qquad - \tau \alpha \, ||\varvec{P}^{(r)}||_{{\text {tr}}} - \tfrac{1}{2} \, ||\varvec{Q}^{(r)} - \varvec{P}^{(r)}||_F^2\\&\quad \le \tau \alpha \, ||\tilde{\varvec{P}}^{(r)} - \varvec{P}^{(r)}||_{{\text {tr}}}\\&\qquad + \tfrac{1}{2} \, \bigl (||\tilde{\varvec{P}}^{(r)} - \varvec{P}^{(r)}||_F + ||\varvec{Q}^{(r)} - \varvec{P}^{(r)}||_F \bigr )^2\\&\qquad - \tfrac{1}{2} \, ||\varvec{Q}^{(r)} - \varvec{P}^{(r)}||_F^2\\&\quad \le \tau \alpha \, ||\tilde{\varvec{P}}^{(r)} - \varvec{P}^{(r)}||_{{\text {tr}}} + \tfrac{1}{2} \, ||\tilde{\varvec{P}}^{(r)} - \varvec{P}^{(r)}||_F^2\\&\qquad + ||\tilde{\varvec{P}}^{(r)} - \varvec{P}^{(r)}||_F ||\varvec{Q}^{(r)} - \varvec{P}^{(r)}||_F\\&\quad \le \tau \alpha \, \sqrt{n} ||\tilde{\varvec{P}}^{(r)} - \varvec{P}^{(r)}||_F + \tfrac{1}{2} \, ||\tilde{\varvec{P}}^{(r)} - \varvec{P}^{(r)}||_F^2\\&\qquad + ||\tilde{\varvec{P}}^{(r)} - \varvec{P}^{(r)}||_F ||\varvec{Q}^{(r)} - \varvec{P}^{(r)}||_F. \end{aligned}$$

Using

$$\begin{aligned} \varvec{P}^{(r)}&= {\text {prox}}_{\tau \alpha \mathscr {R}}(\varvec{Q}^{(r)}) = {\text {proj}}_{\mathscr {C}} (\varvec{Q}^{(r)} - \tau \alpha \varvec{I}_n)\\&= {\text {proj}}_{\mathscr {C}} ((\varvec{Q}^{(r)} - \tau \alpha \varvec{I}_n)_{\succeq \varvec{0}}), \end{aligned}$$

the non-expansiveness of the projection and assuming without loss of generality that $E_r \le 1$, we conclude

$$\begin{aligned}&F(\tilde{\varvec{P}}^{(r)}) - F(\varvec{P}^{(r)})\\&\quad \le \tau \alpha \sqrt{n} E_r + \tfrac{1}{2} \, E_r^2 + E_r \, (||\varvec{Q}^{(r)}||_F + ||\varvec{P}^{(r)}||_F)\\&\quad \le C E_r (||\varvec{Q}^{(r)}||_F + \sqrt{d}), \quad C >0. \end{aligned}$$

Thus, $\tilde{\varvec{P}}^{(r)}$ fulfills the definition of the type-one approximation with precision $C E_r (||\varvec{Q}^{(r)}||_F + \sqrt{d})$, see [39, p. 385]. Since the square roots of the precisions are summable, [39, Thm. 2] ensures the convergence of the inexactly performed primal-dual iteration to a saddle-point of the Lagrangian in (13) and thus to a solution $\hat{\varvec{P}}$ of rreaper. $\square$

## Performance Analysis

Inspired by ideas of Lerman et al. [27], we examine the performance analysis of rreaper. To this end, we assume that the ‘ideal’ subspace *L* of the given data $\varvec{x}_k \in \mathbb {R}^n$, $k=1,\ldots ,N$ has dimension $d_L \le d$. As in [27], we determine the best fit of the data by two measures: the first one is the *distance of the data from the subspace*

$$\begin{aligned} \mathscr {R}_L = \mathscr {R}_L (\varvec{X}) :=\Vert (\varvec{I}_n - \varvec{\varPi }_L) \varvec{X} \Vert _{2,1}, \end{aligned}$$

where $\varPi _L$ denotes the orthogonal projector onto *L*. For the second measure, we assume that the projected data $\{\varvec{\varPi } \varvec{x}_k: k=1,\ldots ,N \}$, $N \ge d_L$, form a *frame* in *L* meaning that there exist constants $0< c_L \le C_L < \infty$ such that

$$\begin{aligned} c_L \le \sum _{k=1}^N |\langle \varvec{u},\varvec{\varPi }_L \varvec{x}_k\rangle |^2 = \sum _{k=1}^N |\langle \varvec{u},\varvec{x}_k\rangle |^2 \le C_L \end{aligned}$$

for all $\varvec{u} \in L$ with $\Vert u\Vert _2 = 1$. In order to recover the entire subspace *L*, the data have obviously to cover each direction in *L* with sufficiently many data points. This well-localization of the data is measured by the *permeance statistic*

23 $$\begin{aligned} \mathscr {P}_L = \mathscr {P}_L(\varvec{X}) :=\min _{\begin{array}{c} \varvec{u} \in L\\ ||\varvec{u}||=1 \end{array}} \sum _{k=1}^N |\langle \varvec{u},\varvec{x}_k\rangle | \end{aligned}$$

which can be seen as $\ell _1$ counterpart of the lower frame bound. Clearly, $\mathscr {P}_L$ becomes large if all direction in *L* are uniformly covered by the data. The lower frame bound and the permeance statistic come into the play in the following lemma, compared with [27, Sect. A2.3].

### Lemma 2

Let $\varvec{\varPi }_L$ be the orthogonal projector onto a subspace *L* of $\mathbb {R}^n$ of dimension $d_L$ and $\varvec{x}_k \in \mathbb {R}^n$, $k=1,\ldots ,N$, $N \ge d_L$ which form the columns of the matrix $\varvec{X}$. Then, for any $\varvec{A} \in \mathbb {R}^{n,n}$, the following relations hold true:

24 $$\begin{aligned} \Vert \varvec{A} \varvec{\varPi }_L \varvec{X}\Vert _{2,2} \ge c_L \Vert \varvec{A} \varvec{\varPi }_L \Vert _F, \end{aligned}$$

and

25 $$\begin{aligned} \Vert \varvec{A} \varvec{\varPi }_L \varvec{X}\Vert _{2,1} \ge \mathscr {P}_L \Vert \varvec{A} \varvec{\varPi }_L \Vert _F \ge \tfrac{1}{ \sqrt{d_L} } \mathscr {P}_L \Vert \varvec{A} \varvec{\varPi }_L \Vert _{{\text {tr}}}. \end{aligned}$$

### Proof

We restrict our attention to (25). The relation (24) follows similar lines. Let $\varvec{A} \, \varvec{\varPi }_L$ have the singular value decomposition $\varvec{A} \, \varvec{\varPi }_L = \varvec{U} \varvec{\varSigma }\varvec{V}^\mathrm {T}$, where the singular values $\sigma _k$, $k=1,\ldots ,n$ are in descending order and $\sigma _{d_L+1}= \ldots = \sigma _n = 0$ and we can arrange $\varvec{V}$ such that the transpose of the first $d_L$ rows of $\varvec{V}$ belong to *L*. Then it holds

$$\begin{aligned} \Vert \varvec{A} \varvec{\varPi }_L\Vert _F^2 = \sum _{k=1}^{d_L} \sigma _k^2. \end{aligned}$$

Using orthogonality of $\varvec{U}$ and concavity of the square root function, we obtain

$$\begin{aligned} \Vert \varvec{A} \varvec{\varPi }_L X\Vert _{2,1}&= \Vert \varvec{U} \varvec{\varSigma }\varvec{V}^\mathrm {T}\varvec{X} \Vert _{2,1} = \Vert \varvec{\varSigma }\varvec{V}^\mathrm {T}\varvec{X} \Vert _{2,1}\\&= \sum _{k=1}^N \left( \sum _{j=1}^{d_L} \sigma _j^2 \langle \varvec{v}_j,\varvec{x}_k \rangle ^2 \right) ^\frac{1}{2} \\&= \Vert \varvec{A} \varvec{\varPi }_L\Vert _F \, \sum _{k=1}^N \left( \sum _{j=1}^{d_L} \frac{\sigma _j^2}{\Vert \varvec{A} \varvec{\varPi }_L\Vert _F^2} \langle v_j,x_k \rangle ^2 \right) ^\frac{1}{2}\\&\ge \Vert \varvec{A} \varvec{\varPi }_L\Vert _F \, \sum _{k=1}^N \sum _{j=1}^{d_L} \frac{\sigma _j^2}{\Vert \varvec{A} \varvec{\varPi }_L\Vert _F^2} |\langle \varvec{v}_j,\varvec{x}_k \rangle |\\&= \Vert \varvec{A} \varvec{\varPi }_L\Vert _F \, \sum _{j=1}^{d_L} \frac{\sigma _j^2}{\Vert \varvec{A} \varvec{\varPi }_L\Vert _F^2} \sum _{k=1}^N |\langle \varvec{v}_j, \varvec{x}_k \rangle |\\&\ge \mathscr {P}_L \, \Vert \varvec{A} \varvec{\varPi }_L\Vert _F . \end{aligned}$$

The second estimate in (25) follows by (1). $\square$

Now we can estimate the reconstruction error of rreaper.

### Theorem 2

Let $\varvec{\varPi }_L$ be the orthogonal projector onto a subspace *L* of $\mathbb {R}^n$ of dimension $d_L$ and $\varvec{x}_k \in \mathbb {R}^n$, $k=1,\ldots ,N$, $N \ge d_L$ such that their projections onto *L* form a frame of *L*. Define $\mathscr {P}_L$ by (23) and set $\gamma _L :=\frac{1}{2\sqrt{2 d_L}} \mathscr {P}_L$. Let $\hat{\varvec{P}}$ be the solution of (9) and $\hat{\varvec{\varPi }}$ the projection of $\hat{\varvec{P}}$ onto $\mathscr {O}_d$. Then, for $\alpha \le 2\gamma _L$ and $d \ge d_L$, the reconstruction error is bounded by

26 $$\begin{aligned} ||\hat{\varvec{\varPi }} - \varvec{\varPi }_L||_{{\text {tr}}} \le \min \left\{ \frac{6 \mathscr {R}_L}{\gamma _L- |\gamma _L - \alpha |}, \frac{6 \mathscr {R}_L + 2 d |\alpha - \gamma _L|}{\gamma _L} \right\} . \end{aligned}$$

### Proof

Since $\hat{\varvec{P}}$ is a minimizer of (9), we obtain

27 $$\begin{aligned} 0&\le \Vert (\varvec{I}_n - \varvec{\varPi }_L)\varvec{X}\Vert _{2,1} - \Vert (\varvec{I}_n - \hat{\varvec{P}}) \varvec{X}\Vert _{2,1} \nonumber \\&\quad + \alpha (\Vert \varvec{\varPi }_L\Vert _{{\text {tr}}} - \Vert \hat{\varvec{P}}\Vert _{{\text {tr}}}) \end{aligned}$$

28 $$\begin{aligned}&= \mathscr {R}_L - \Vert (\varvec{I}_n - \hat{\varvec{P}}) \varvec{X}\Vert _{2,1} + \alpha (d_L - \Vert \hat{\varvec{P}}\Vert _{{\text {tr}}}) \nonumber \\&\le \mathscr {R}_L + \Vert (\varvec{I}_n - \hat{\varvec{P}}) (\varvec{I}_n -\varvec{\varPi }_L) \varvec{X}\Vert _{2,1}\nonumber \\&\quad - \Vert (\varvec{I}_n - \hat{\varvec{P}}) \varvec{\varPi }_L \varvec{X}\Vert _{2,1} + \alpha (d_L - \Vert \hat{\varvec{P}}\Vert _{{\text {tr}}})\nonumber \\&= \mathscr {R}_L + \Vert \big (\varvec{I}_n - (\hat{\varvec{P}} - \varvec{\varPi }_L) \big ) (\varvec{I}_n -\varvec{\varPi }_L) \varvec{X}\Vert _{2,1} \nonumber \\&\quad - \Vert (\varvec{I}_n - \hat{\varvec{P}}) \varvec{\varPi }_L \varvec{X}\Vert _{2,1} + \alpha (d_L - \Vert \hat{\varvec{P}}\Vert _{{\text {tr}}})\nonumber \\&\le \bigl ( 1 + \Vert \varvec{I}_n - (\hat{\varvec{P}} - \varvec{\varPi }_L)\Vert _2 \bigr ) \mathscr {R}_L - \Vert (\varvec{I}_n - \hat{\varvec{P}}) \varvec{\varPi }_L \varvec{X}\Vert _{2,1} \nonumber \\&\quad + \alpha (d_L - \Vert \hat{\varvec{P}}\Vert _{{\text {tr}}}) \end{aligned}$$

It remains to estimate

$$\begin{aligned} \Vert (\varvec{I}_n - \hat{\varvec{P}}) \varvec{\varPi }_L \varvec{X}\Vert _{2,1} = \Vert (\hat{\varvec{P}} - \varvec{\varPi }_L) \varvec{\varPi }_L \varvec{X}\Vert _{2,1} \end{aligned}$$

from below. To this end, we decompose $\varvec{\varDelta }:= \hat{\varvec{P}} - \varvec{\varPi }_L$ as

$$\begin{aligned} \varvec{\varDelta }&= \underbrace{ \varvec{\varPi }_{L} \varvec{\varDelta }\varvec{\varPi }_{L} }_{\varvec{\varDelta }_1} + \underbrace{ (\varvec{I}_n - \varvec{\varPi }_L) \varvec{\varDelta }\varvec{\varPi }_{L} }_{\varvec{\varDelta }_2} + \underbrace{ \varvec{\varPi }_{L} \varvec{\varDelta }(\varvec{I}_n - \varvec{\varPi }_L) }_{\varvec{\varDelta }_2^\mathrm {T}}\\&\quad + \underbrace{ (\varvec{I}_n - \varvec{\varPi }_L) \varvec{\varDelta }(\varvec{I}_n - \varvec{\varPi }_L) }_{\varvec{\varDelta }_3}. \end{aligned}$$

Since $\varvec{0}_{n,n} \preceq \hat{ \varvec{P}} \preceq \varvec{I}_n$, we obtain be conjugation with $\varvec{\varPi }_L$, resp. $I_n - \varvec{\varPi }_L$ that $\varvec{\varDelta }_1 \preceq \varvec{0}_{n,n}$ and $\varvec{0}_{n,n} \preceq \varvec{\varDelta }_3$, so that $\Vert \varvec{\varDelta }_1 \Vert _{{\text {tr}}} = -{\text {tr}}{\varvec{\varDelta }_1}$ and $\Vert \varvec{\varDelta }_3 \Vert _{{\text {tr}}} = {\text {tr}}{\varvec{\varDelta }_3}$. Then we conclude

$$\begin{aligned} {\text {tr}}(\varvec{\varDelta })&= {\text {tr}}(\hat{\varvec{P}}) - d_L = {\text {tr}}{\varvec{\varDelta }_1} + 2 {\text {tr}}{\varvec{\varDelta }_2} + {\text {tr}}{\varvec{\varDelta }_3}\\&= {\text {tr}}{\varvec{\varDelta }_1} + {\text {tr}}{\varvec{\varDelta }_3} = \Vert \varvec{\varDelta }_3 \Vert _{{\text {tr}}} - \Vert \varvec{\varDelta }_1 \Vert _{{\text {tr}}}, \end{aligned}$$

which implies

$$\begin{aligned} ||\varvec{\varDelta }||_{{\text {tr}}}&\le ||\varvec{\varDelta }_1||_{{\text {tr}}} +||\varvec{\varDelta }_2||_{{\text {tr}}} + ||\varvec{\varDelta }_2^\mathrm {T}||_{{\text {tr}}} + ||\varvec{\varDelta }_3||_{{\text {tr}}}\\&= 2 ||\varvec{\varDelta }_1||_{{\text {tr}}} + 2 ||\varvec{\varDelta }_2||_{{\text {tr}}} + {\text {tr}}(\hat{\varvec{P}})-d_L. \end{aligned}$$

Now we can estimate the last summand by (1) and Lemma 2 as

29 $$\begin{aligned} \Vert \varvec{\varDelta }\varvec{\varPi }_L \varvec{X}\Vert _{2,1}&= \Vert \varvec{\varPi }_{L} \varvec{\varDelta }\varvec{\varPi }_L \varvec{X} + (\varvec{I}_n - \varvec{\varPi }_L) \varvec{\varDelta }\varvec{\varPi }_L \varvec{X}\Vert _{2,1} \nonumber \\&= \sum _{k=1}^N \left( \Vert \varvec{\varDelta }_1 \varvec{x}_k\Vert _2^2 + \Vert \varvec{\varDelta }_2 \varvec{x}_k\Vert _2^2\right) ^\frac{1}{2}\nonumber \\&\ge \frac{1}{\sqrt{2}} \sum _{k=1}^N \left( \Vert \varvec{\varDelta }_1 \varvec{x}_k\Vert _2 +\Vert \varvec{\varDelta }_2 \varvec{x}_k\Vert _2 \right) \nonumber \\&\ge \frac{1}{ \sqrt{2 d_L}} \mathscr {P}_L \left( \Vert \varvec{\varDelta }_1\Vert _{{\text {tr}}} + \Vert \varvec{\varDelta }_2 \Vert _{{\text {tr}}} \right) \nonumber \\&\ge \frac{1}{2\sqrt{2 d_L}} \mathscr {P}_L \left( \Vert \varvec{\varDelta }\Vert _{{\text {tr}}} + d_L - \Vert \hat{\varvec{P}}\Vert _{{\text {tr}}} \right) . \end{aligned}$$

By (28) and (29), we obtain

30 $$\begin{aligned} 0&\le (1 + \Vert \varvec{I}_n - (\hat{\varvec{P}} - \varvec{\varPi }_L)\Vert _2) \, \mathscr {R}_L \nonumber \\&\quad + (\alpha - \gamma _L) (d_L - \Vert \hat{\varvec{P}}\Vert _{{\text {tr}}}) - \gamma _L \Vert \hat{\varvec{P}} - \varvec{\varPi }_L\Vert _{{\text {tr}}} \nonumber \\&\le (1 + \Vert \varvec{I}_n - (\hat{\varvec{P}} - \varvec{\varPi }_L)\Vert _2) \, \mathscr {R}_L \nonumber \\&\quad + |\alpha - \gamma _L| |d_L - \Vert \hat{\varvec{P}}\Vert _{{\text {tr}}}| - \gamma _L \Vert \hat{\varvec{P}} - \varvec{\varPi }_L\Vert _{{\text {tr}}} . \end{aligned}$$

Using the triangular inequality $\Vert \hat{\varvec{P}} - \varvec{\varPi }_L\Vert _{{\text {tr}}} \ge | d_L - \Vert \hat{\varvec{P}}\Vert _{{\text {tr}}}|$, we get

31 $$\begin{aligned} \begin{aligned} 0&\le (1 + \Vert \varvec{I}_n - (\hat{\varvec{P}} - \varvec{\varPi }_L)\Vert _2) \, \mathscr {R}_L \\&\quad + \left( |\alpha - \gamma _L| - \gamma _L \right) \ \Vert \hat{\varvec{P}} - \varvec{\varPi }_L\Vert _{{\text {tr}}} . \end{aligned} \end{aligned}$$

We next employ the estimate

32 $$\begin{aligned} \Vert \varvec{I}_n - (\hat{\varvec{P}} - \varvec{\varPi }_L)\Vert _2 \le \Vert \varvec{I}_n - \hat{\varvec{P}}\Vert _2 + \Vert \varvec{\varPi }_L\Vert _2 \ \le 2, \end{aligned}$$

which results in

$$\begin{aligned} \Vert \hat{\varvec{P}} - \varvec{\varPi }_L\Vert _{{\text {tr}}} \le \frac{3 \mathscr {R}_L}{\gamma _L - |\gamma _L - \alpha |} \end{aligned}$$

if $\alpha < 2 \gamma _L$. Alternatively, we apply (32) in (30) together with $|d_L - \Vert \hat{\varvec{P}}\Vert _{{\text {tr}}}| \le d$, since $d_L \le d$, and obtain

$$\begin{aligned} \Vert \hat{\varvec{P}} - \varvec{\varPi }_L\Vert _{{\text {tr}}} \le \frac{3 \mathscr {R}_L + d |\alpha - \gamma _L|}{\gamma _L}. \end{aligned}$$

The final assertion follows by

$$\begin{aligned} \Vert \hat{\varvec{\varPi }} - {\varvec{\varPi }}_L\Vert _{{\text {tr}}} \le \Vert \hat{\varvec{\varPi }} - \hat{\varvec{P}}\Vert _{{\text {tr}}} +\Vert \hat{\varvec{P}} - {\varvec{\varPi }}_L\Vert _{{\text {tr}}} \le 2 \Vert \hat{\varvec{P}} - {\varvec{\varPi }}_L\Vert _{{\text {tr}}}. \end{aligned}$$

$$\square $$

The error depends basically on the quotient of the distance of the normalized data from the subspace and the permeance statistics, where the normalization is indirectly contained in the quotient. For $\alpha = \gamma _L$, the terms of the minimum in (26) are the same and both terms become small; the second term is preferred for $\alpha$ near zero or $2 \gamma _L$. Compared to our experimental results, the estimate is in general too pessimistic.

### Remark 5

In [27], the following estimate was given for the original reaper

33 $$\begin{aligned}&||\hat{\varvec{\varPi }} - \varvec{\varPi }_L||_{{\text {tr}}}\nonumber \\&\quad \le 16 \sqrt{d_L} \mathscr {R}_L(\varvec{X}_{\mathrm {in}})\nonumber \\&\qquad \Big /\Bigl ( \mathscr {P}_L(\varvec{X}_{\mathrm {in}}) - 4 \sqrt{d_L} \mathscr {R}_L(\varvec{X}_{\mathrm {in}})\nonumber \\&\qquad - 4 \sqrt{d_L} ||\varvec{X}_{\mathrm {out}}|| \, ||((\varvec{I}_n - \varvec{\varPi }_L) \varvec{X}_{\mathrm {out}})^\sim ||\Bigr )_+, \end{aligned}$$

where the data points $\varvec{X} = [\varvec{X}_{\mathrm {in}} | \varvec{X}_{\mathrm {out}}]$ were divided into inliers $\varvec{X}_{\mathrm {in}} \in \mathbb {R}^{n,N_{\mathrm {in}}}$ and outliers $\varvec{X}_{\mathrm {out}} \in \mathbb {R}^{n,N_{\mathrm {out}}}$ and $(\cdot )^\sim$ normalizes the columns of a matrix one. Following the lines of the proof in those paper, we would obtain the estimate

34 $$\begin{aligned}&||\hat{\varvec{\varPi }} - \varvec{\varPi }_L||_{{\text {tr}}} \nonumber \\&\quad \le \Bigl ( 16 \sqrt{d_L} \mathscr {R}_L(\varvec{X}_{\mathrm {in}}) + 8 \alpha d_L \sqrt{d_L} \Bigr ) \nonumber \\&\qquad \Big /\Bigl ( \mathscr {P}_L(\varvec{X}_{\mathrm {in}}) - 4 \sqrt{d_L} \mathscr {R}_L(\varvec{X}_{\mathrm {in}}) \nonumber \\&\qquad - 4 \sqrt{d_L} ||\varvec{X}_{\mathrm {out}}|| \, ||((\varvec{I}_n - \varvec{\varPi }_L) \varvec{X}_{\mathrm {out}})^\sim ||\Bigr )_+ \end{aligned}$$

for rreaper. Therefore, rreaper inherits the robustness from reaper as well as the theoretical guarantees for certain data models. However, in our numerical examples, we realized that the above estimate is often not defined due to division by zero, especially in the presents of already small inlier noise, i.e., if the columns of $\varvec{X}_{\mathrm {in}}$ are only near the subspace *L*. Moreover, it is not clear how the division into in- and outliers can be achieved for real data sets. In our estimate (26), we do not distinguish between in- and outliers. Using alternatively $\Vert \varvec{I}_n - (\hat{\varvec{P}} - \varvec{\varPi }_L)\Vert _2 \le 1 + \Vert \hat{\varvec{P}} - \varvec{\varPi }_L\Vert _{{\text {tr}}}$ in (31), we would obtain the estimate

$$\begin{aligned} \Vert \hat{\varvec{\varPi }} - \varvec{\varPi }_L\Vert _{{\text {tr}}} \le \frac{8 \sqrt{2 d_L} \mathscr {R}_L}{\left( \mathscr {P}_L - 2 \sqrt{2 d_L}\mathscr {R}_L - |\mathscr {P}_L - 2 \sqrt{2 d_L} \alpha |\right) _+}. \end{aligned}$$

## Incorporating the Offset

So far, we have assumed that the offset $\varvec{b}$ in the robust PCA problem is given, so that we can just search for a low-dimensional linear subspace which represents the data well. While in the classical PCA (4), the affine subspace always passes through the mean value (5) of the data, it is not clear which value $\varvec{b}$ must be chosen in order to minimize

35 $$\begin{aligned} \begin{aligned}&E(\varvec{A},\varvec{b}) :=\sum _{i=1}^N \Vert (\varvec{A} \varvec{A}^\mathrm {T}- \varvec{I}_n )(\varvec{b}-\varvec{x}_i)\Vert _2\\&\quad \mathrm {subject } \; \mathrm{to} \quad \varvec{A}^\mathrm {T}\varvec{A} = \varvec{I}_d. \end{aligned} \end{aligned}$$

Clearly, if $(\hat{\varvec{A}}, \hat{\varvec{b}})$ is a minimizer of *E*, then, for every $\varvec{b} \in {\text {ran}}(\hat{\varvec{A}})$, also $(\hat{\varvec{A}}, \hat{\varvec{b}} + \varvec{b})$ is a minimizer.

A common choice for the offset is the *geometric median* of the data points defined by

$$\begin{aligned} \hat{\varvec{b}} :=\mathop {{\text {argmin}}}\limits _{\varvec{b} \in \mathbb {R}^n} \sum _{k=1}^N \Vert \varvec{b} - \varvec{x}_k\Vert _2, \end{aligned}$$

which can be computed, e.g., by the Weiszfeld algorithm and its generalizations, see, e.g., [2, 36, 42, 49]. Other choices arising, e.g., from Tyler’s *M*-estimator or other robust statistical approaches [19, 24, 26, 33, 47], were proposed in the literature. However, they do in general not minimize (35) as the following example from [35] shows: given three points in $\mathbb {R}^n$ which form a triangle with angles smaller than 120$^\circ$, the geometric median is the point in the inner of the triangle from which the points can be seen under an angle of 120$^\circ$. In contrast, the line ($d=1$) having smallest distance from the three points is the one which passes through those two points with the largest distance from each other.

In the following, we show that there always exists an optimal hyperplane of dimension $d=n-1$ in $\mathbb {R}^n$ determined by a minimizer of *E* in (35) that contains at least *n* data points. Further, if the number *N* of data points is odd, then *every* optimal hyperplane contains at least *n* data points. Recall that the hyperplane spanned by the columns of $\varvec{A} = (\varvec{a}_1|\ldots |\varvec{a}_{n-1}) \in \mathrm {St}(n,n-1)$ with offset $\varvec{b}$ is given by

$$\begin{aligned} \{\varvec{x} = \varvec{A} \varvec{t} + \varvec{b}: \varvec{t} \in \mathbb {R}^{n-1} \} = \{\varvec{x} \in \mathbb {R}^n: \langle \varvec{a}^\perp , \varvec{x} \rangle = \beta \}, \end{aligned}$$

where $\varvec{a}^\perp \perp \varvec{a}_i$, $i=1,\ldots ,n-1$ is a unit normal vector of the hyperplane, which is uniquely determined up to its sign and $\langle \varvec{a}^\perp ,\varvec{b} \rangle = - \beta$.

The following lemma describe the placement of the data points with respect to the halfspaces determined by a minimizing hyperplane.

### Lemma 3

Let $\varvec{x}_k \in \mathbb {R}^n$, $k=1,\ldots ,N$. Let $(\hat{\varvec{A}},\hat{\varvec{b}})$ be a minimizer of *E* and $N=M + M_+ + M_-$ with

$$\begin{aligned}&M :=|\{\varvec{x}_k: \langle \hat{\varvec{a}}^\perp , \varvec{x}_i \rangle = \hat{\beta }\}|,\\&M_+ :=|\{\varvec{x}_k: \langle \hat{\varvec{a}}^\perp , \varvec{x}_i \rangle > \hat{\beta }\}|,\\&M_- :=|\{\varvec{x}_k: \langle \hat{\varvec{a}}^\perp , \varvec{x}_i \rangle < \hat{\beta }\}|. \end{aligned}$$

Then it holds $|M_+ - M_-| \le M$. In particular, it holds $M \ge 1$ if *N* is odd. Also for even *N* there exists a minimizing hyperplane with $\hat{\varvec{b}} = \varvec{x}_k$ for some $k \in \{1,\ldots ,N\}$.

### Proof

W.l.o.g. assume that $M_+ \ge M_-$. If $M_+ = 0$, then all data points are in the minimizing hyperplane and we are done. Otherwise, the value

$$\begin{aligned} \varepsilon :=\min \{ \langle \hat{\varvec{a}}^\perp , \varvec{x}_k \rangle > 0: k=1,\ldots ,N \} \end{aligned}$$

is positive, and we consider the shifted hyperplane $\{\varvec{x} \in \mathbb {R}^n: \langle \varvec{a}^\perp , \varvec{x} \rangle = \beta + \varepsilon \}$, which contains at least one data point. The sum of the distances of the data points from this hyperplane is

$$\begin{aligned} E(\hat{\varvec{A}},\hat{\varvec{b}}) - \varepsilon M_+ + \varepsilon (M+M_-). \end{aligned}$$

Since $(\hat{\varvec{A}},\hat{\varvec{b}})$ is a minimizer of *E*, this implies that $M_+ \le M + M_-$. If $M = 0$, then $M_- = M_+$ and the shifted hyperplane is also minimizing. However, this case cannot appear for odd *N* so that $M \ge 1$ for odd *N*. This finishes the proof. $\square$

### Theorem 3

Let $\varvec{x}_\ell \in \mathbb {R}^n$, $\ell =1,\ldots ,N$. Then there exists a minimizer $(\hat{\varvec{A}},\hat{\varvec{b}})$ of *E* such that the corresponding minimizing hyperplane contains at least *n* data points. If *N* is odd, every minimizing hyperplane contains at least *n* data points.

### Proof

By Lemma 3, there exists a data point $\varvec{x}_\ell$ such that $(\hat{\varvec{A}}, \hat{\varvec{b}})$ with $\hat{\varvec{b}} = \varvec{x}_\ell$ is a minimizer of *E* and for odd *N* every minimizing hyperplane passes through a data point. Let $\hat{\varvec{a}}^\perp$ be a unit vector orthogonal to the columns of $\hat{\varvec{A}}$. Set $\varvec{y}_k :=\varvec{x}_k - \varvec{x}_\ell$, $k=1,\ldots ,N$. Next, we show: if the subspace normal to $\hat{\varvec{a}}^\perp$ contains *M* linearly independent vectors $\varvec{y}_1, \dots , \varvec{y}_M$ with $0 \le M \le n-2$, then exactly one of the following situations apply. (i) The remaining vectors $\varvec{y}_k$ with $k=M+1, \dots , N$ are linearly dependent from the first *M* vectors and thus in the same linear subspace $\mathrm {span} \{\varvec{y}_k: k=1,\ldots ,M\}$. (ii) We find a further independent vector, say $\varvec{y}_{M+1}$, contained in the subspace normal to $\hat{\varvec{a}}^\perp$ such that we can increase *M* to $M+1$. Repeating this argumentation until $M = n-2$, we are done since $\varvec{y}_k + \varvec{x}_\ell$, $k=1,\ldots ,n-1$ and $\varvec{x}_\ell$ itself are in the subspace corresponding to $(\hat{\varvec{A}}, \hat{\varvec{b}})$.

Because the vectors $\varvec{y}_k$ with $k=1,\ldots ,M$ are independent and are contained in the subspace normal to $\hat{\varvec{a}}^\perp$ by assumption, there exists a matrix

$$\begin{aligned} \varvec{B} :=(\varvec{u}_1|\ldots |\varvec{u}_M| \varvec{u}_{M+1}|\ldots | \varvec{u}_{n-1}) \in \mathrm {St}(n,n-1), \end{aligned}$$

with ${\text {ran}}(\varvec{A}) = {\text {ran}}(\varvec{B})$, whose first columns have the same span as $\varvec{y}_1, \dots , \varvec{y}_M$, i.e.,

$$\begin{aligned} \mathrm {span}\{\varvec{u}_k:k=1,\ldots ,M\} = \mathrm {span}\{\varvec{y}_k:k=1,\ldots ,M\}. \end{aligned}$$

This especially implies $\varvec{y}_k \perp \varvec{u}_\ell$ for $k = 1, \dots , M$ and $\ell = M+1, \dots , n-1$. Note that the normal unit vector of ${\text {ran}}(\varvec{B})$ is also $\hat{\varvec{a}}^\perp$, and that the objectives coincides, i.e.,

36 $$\begin{aligned} \sum _{k=1}^N \Vert ( \hat{\varvec{A}} \hat{\varvec{A}}^\mathrm {T}- I_n) \varvec{y}_k \Vert _2 = \sum _{k=1}^N \Vert ( \varvec{B} \varvec{B}^\mathrm {T}- I_n) \varvec{y}_k \Vert _2. \end{aligned}$$

Now, let the matrix-valued function

$$\begin{aligned} \phi _{\varvec{B}}: [-\pi ,\pi ) \rightarrow \mathrm {St}(n,n-1) \end{aligned}$$

be defined by

$$\begin{aligned} \phi _{\varvec{B}}(\alpha ) := \varvec{Q}_{\varvec{B}} \, \varvec{R}(\alpha ) \, \varvec{C}, \end{aligned}$$

where the three building factors are given by

$$\begin{aligned} \varvec{Q}_{\varvec{B}}&:=(\varvec{B}|\hat{\varvec{a}}^\perp ), \\ \varvec{R}(\alpha )&:=\left( \begin{array}{ccc} \varvec{I}_{n-2} &{}\varvec{0}_{n-2}&{}\varvec{0}_{n-2}\\ \varvec{0}_{n-2}^\mathrm {T}&{}\cos (\alpha )&{}\sin (\alpha )\\ \varvec{0}_{n-2}^\mathrm {T}&{}-\sin (\alpha )&{}\cos (\alpha ) \end{array} \right) ,\\ \varvec{C}&:=\begin{pmatrix} \varvec{I}_{n-1}\\ \varvec{0}_{n-1}^\mathrm {T}\end{pmatrix}. \end{aligned}$$

Figuratively, the function $\phi _{\varvec{B}}$ takes the orthonormal columns of $\varvec{B}$ and rotates the last vector $\varvec{u}_{n-1}$ by the angle $\alpha$ in the plane spanned by $\varvec{u}_{n-1}$ and $\hat{\varvec{a}}^\perp$. Clearly, we have $\phi _{\varvec{B}}(0) = \varvec{B}$. Due to (36), the function

$$\begin{aligned} F(\alpha ) = \sum _{k=1}^N \Vert ( \phi _{\varvec{B}}(\alpha ) \phi _{\varvec{B}}(\alpha )^\mathrm {T}- \varvec{I}_n) \varvec{y}_k \Vert _2 = \sum _{k=1}^N f_k(\alpha ) \end{aligned}$$

has moreover a minimum in $\alpha = 0$. For the summands of *F*, we obtain

$$\begin{aligned} f_k(\alpha )&= \Vert ( \phi _{\varvec{B}}(\alpha ) \phi _{\varvec{B}}(\alpha )^\mathrm {T}- \varvec{I}_n ) \, \varvec{y}_k \Vert _2\\&= \Vert ( \varvec{Q}_{\varvec{B}} \, \varvec{R}(\alpha ) \, \varvec{C} \, \varvec{C}^\mathrm {T}\, \varvec{R}(\alpha )^\mathrm {T}\, \varvec{Q}_{\varvec{B}}^\mathrm {T}- \varvec{I}_n ) \, \varvec{y}_k \Vert _2\\&= \Vert ( \varvec{C} \, \varvec{C}^\mathrm {T}\, \varvec{R}(\alpha )^\mathrm {T}\, \varvec{Q}_{\varvec{B}}^\mathrm {T}- \varvec{R}(\alpha )^\mathrm {T}\, \varvec{Q}_{\varvec{B}}^\mathrm {T}) \, \varvec{y}_k \Vert _2\\&= \Vert (\varvec{C} \, \varvec{C}^\mathrm {T}- \varvec{I}_n) \varvec{R}(\alpha )^\mathrm {T}\, \varvec{Q}_{\varvec{B}}^\mathrm {T}\varvec{y}_k \Vert _2\\&= \left| \sin (\alpha ) \langle \varvec{u}_{n-1}, \varvec{y}_k \rangle + \cos (\alpha ) \langle \hat{\varvec{a}}^\perp , \varvec{y}_k \rangle \right| , \end{aligned}$$

since $\varvec{Q}_{\varvec{B}}$ and $\varvec{R}(\alpha )$ are orthogonal by construction. Hence, we get

$$\begin{aligned} F(\alpha ) = \sum _{k=M+1}^N \left| \sin (\alpha ) \langle \varvec{u}_{n-1}, \varvec{y}_k \rangle + \cos (\alpha ) \langle \hat{\varvec{a}}^\perp , \varvec{y}_k \rangle \right| . \end{aligned}$$

Here the first *M* summands vanish because of the mentioned orthogonality $\varvec{y}_k \perp \varvec{u}_{n-1}$ and $\varvec{y}_k \perp \hat{\varvec{a}}^\perp$ for $k=1, \dots , M$.

If all remaining given points $\varvec{y}_k$ with $k=M+1,\ldots ,N$ are in $\mathrm {span}\{\varvec{y}_1, \ldots , \varvec{y}_M\}$, then the corresponding remaining summands of $F(\alpha )$ become zero too, and the first situation (i) applies; so we are done.

If this is not the case, consider only those $\varvec{y}_k$ with $k=M+1,\ldots ,N$ that are linearly independent of the $\varvec{y}_k$, $k=1,\ldots ,M$. Let us denote the corresponding non-empty index set by $\mathscr {I}$. Assume that there exists a $k \in \mathscr {I}$ such that $f_k$ is not differentiable in $\alpha = 0$. This is only possible if the argument of the absolute value vanishes implying

$$\begin{aligned} f_k(0) = |\langle \hat{\varvec{a}}^\perp , \varvec{y}_k \rangle | = 0. \end{aligned}$$

Thus, the vector $\varvec{y}_k$ is in the subspace spanned by the columns of $\hat{\varvec{A}}$, and we are done. Otherwise, if $f_k(0) \not = 0$ for all $k\in \mathscr {I}$, then it is differentiable in $\alpha = 0$ and, by straightforward differentiation, we obtain

$$\begin{aligned} f_k''(0) = - f_k(0) < 0. \end{aligned}$$

But then $\alpha = 0$ cannot be a minimizer of *F* which is a contradiction. Hence, this case cannot occur and the proof is complete. $\square$

If the target dimension *d* of the minimizing subspace is strictly less than $n-1$, then it does not have to contain any data point as the following example shows.

### Counterexample 1

(*Lower-dimensional subspace approximation*) Initially, we consider the approximation of some given points in $\mathbb {R}^3$ by an one-dimensional subspace—a line. More precisely, for a fixed $T \gg 1$, we consider the six given points

$$\begin{aligned} (\cos (\phi ), \sin (\phi ), \pm T)^\mathrm {T}\quad \text {with}\quad \phi \in \{0, \nicefrac {2\pi }{3}, \nicefrac {4\pi }{3}\}. \end{aligned}$$

We thus have two well-separated clusters around $(0,0, T)^\mathrm {T}$ and $(0,0,-T)^\mathrm {T}$.

Obviously, the optimal line has somehow to go through each cluster. One possible candidate for the approximation line is simply the axis $\{(0,0,t) : t \in \mathbb {R}\}$, whose distance to the given points is by construction 6—for each cluster 3. Now, assume that the line goes through one given point, say $(1,0,T)^\mathrm {T}$. If *T* is very large, then we can neglect the slope of the line. Only considering the distances within the cluster around $(0,0,T)^\mathrm {T}$, we notice that the distance increases from 3 to approximately $2 \sqrt{3}$. Although the axis is maybe not the optimal line, the distance to the given points is smaller than for a line going through a data point. Therefore, we can conclude that the optimal line has not to contain any given point.

The same construction can be done for arbitrary subspaces of dimension $d < n-1$. For example, consider just the points

$$\begin{aligned} (\cos (\phi ), \sin (\phi ) | \pm T \varvec{e}_k^\mathrm {T})^\mathrm {T}\end{aligned}$$

with

$$\begin{aligned} \phi \in \{0, \nicefrac {2\pi }{3}, \nicefrac {4\pi }{3}\}, k = 1, \dots , d, \end{aligned}$$

where $\varvec{e}_k$ is the *k*th unit vector. Using the same argumentation as above, the distance to the subspace $\mathrm {span} \{ \pm \varvec{e}_k : k=1,\dots , d\}$ is smaller than to any *d*-dimensional subspace containing at least one data point.

## Numerical Examples

In this section, we demonstrate the performance of rreaper by numerical examples implemented in MATLAB$^{\textregistered }$ (R2019b, 64-bit) and calculated using an Intel$^{\copyright }$ Core$^{\mathrm{TM}}$ i7-8700 CPU (6 $\times$ 3.20 GHz) and 32 GiB main memory. For synthetic data, we employ the so-called *haystack model* introduced by Lerman et al. in [27]. The idea is to generate a set of inliers $\varvec{X}_{\mathrm {in}} \in \mathbb {R}^{n,N_{\mathrm {in}}}$ lying near an $d_L$-dimensional subspace—here the subspace spanned by the first $d_L$ unit vectors—and a set of outliers $\varvec{X}_{\mathrm {out}} \in \mathbb {R}^{n,N_{\mathrm {out}}}$ located somewhere in the surrounding space. More precisely, the elements of the in- and outliers are drawn form the Gaussians

$$\begin{aligned}&(\varvec{X}_{\mathrm {in}})_{\ell , k} \sim {\left\{ \begin{array}{ll} \mathscr {N}(0, \sigma _{\mathrm {in}}^2/d_L) &{} \text {if} \; \ell \le d_L,\\ \mathscr {N}(0, \sigma _{\mathrm {noise}}^2 / (n-d_L)) &{} \text {otherwise}, \end{array}\right. } \end{aligned}$$

and

$$\begin{aligned} (\varvec{X}_{\mathrm {out}})_{\ell , k} \sim \mathscr {N}(0, \sigma _{\mathrm {out}}^2/n). \end{aligned}$$

The complete data set then consists of the matrix

$$\begin{aligned} \varvec{X} = [\varvec{X}_{\mathrm {in}} | \varvec{X}_{\mathrm {out}}]. \end{aligned}$$

Further, we compare the results of the matrix-free rreaper with several other robust PCA methods which can be applied to high-dimensional images. More precisely, we consider the following methods:

- *PCA-L1 method* [21] maximizes the L1 dispersion $||\varvec{P} \varvec{X}||_{1,1}$. The method is based on a greedy search algorithm that determines the maximum $\hat{\varvec{P}}$ by finding the rank-one projections to the principal components. The method finds a local maximum, but there is no guarantee to compute the global one. Indeed, the results heavily depends on the start value, which is the reason why we restart the algorithm several times (actually 10 times). Further, in contrast to the other three methods, this one is not rotationally invariant due to the objective function which often results in lower quality.
- *Nested Weiszfeld algorithm* [35] aims to approximate the minimizer of $||(\varvec{I}_n - \varvec{P}) \varvec{X}||_{2,1}$. The projection is determined by a greedy-like method by computing the principle components sequentially. In general, this does not yield a (local) minimizer of $||(\varvec{I}_n - \varvec{P}) \varvec{X}||_{2,1}$.
- *R1-PCA* [9, 34], which minimizes again the data fidelity $||(\varvec{I}_n - \varvec{P}) \varvec{X}||_{2,1}$. The minimization is performed by a gradient descent method on the Grassmannian manifold which is equivalent to a conditional gradient algorithm—also known as Frank–Wolfe algorithm. Convergence to a (local) minimizer is only guaranteed under certain assumptions on the anchor set.
- *Fast Median Subspace method (FMS)* [25] is a heuristic method to minimize the energy $||(\varvec{I}_n - \varvec{P}) \varvec{X}||_{2,p} := \sum _{k=1}^N ||(\varvec{I}_n - \varvec{P}) \varvec{x}_k||_2^p$ with $0< p < 2$ iteratively. For this, a sequence of subspaces is constructed on the basis of weighted PCAs. FMS always converge to a stationary point (or to a continuum of stationary points), which are usually (local) minimizers of the energy functional.
- *Geodesic Gradient Descent (GGD)* [31] intends to minimizes $||(\varvec{I}_n - \varvec{P}) \varvec{X}||_{2,1}$ by a geodesic descent performed on the Grassmannian. Under certain conditions on the problem and starting value, this method linear converges to the underlying unknown subspace.

### (2,1)-Norm versus Frobenius Norm

This example with simple synthetic data will show that the (2,1)-norm in the data term is more robust against outliers than the Frobenius norm

$$\begin{aligned} \left( \sum _{k=1}^N ||(\varvec{P} - \varvec{I}_n) \, \varvec{x}_k||_2^2 \right) ^\frac{1}{2} = ||\varvec{P} \varvec{X} - \varvec{X}||_F. \end{aligned}$$

For the Frobenius norm here abbreviated as *F*-norm, we have only to replace the projection onto $\mathfrak B_{2, \infty }$ with the projection to the Frobenius norm ball

$$\begin{aligned} {\text {proj}}_{\mathfrak {B}_{F}}(\varvec{Y}) = \frac{\varvec{Y}}{[||\varvec{Y}|| - 1]_+ + 1}. \end{aligned}$$

**Fig. 1:**
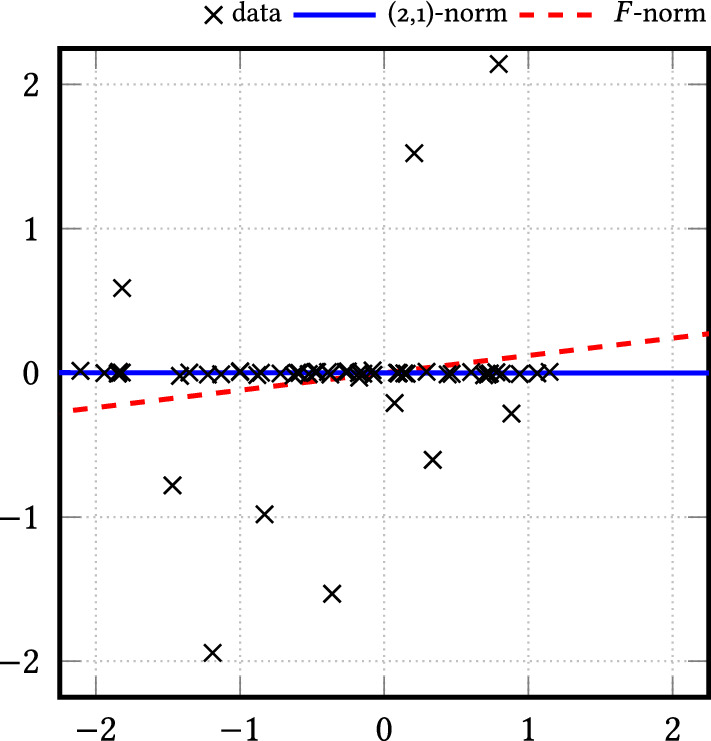
Performance of rreaper with (2,1)-norm and Frobenius norm in the data fidelity term, respectively. The first one appears to be more robust against outliers

We want to recover a line in the plane. Since this recovery problem is invariant under rotations, we restrict ourselves to $\mathrm {span} \{ (1,0)^\mathrm {T}\}$. The data are generated randomly using the haystack model and consist of 50 points near the considered axis—we added a small amount of noise in the second coordinate—and of 10 outliers located somewhere in the plane, see Fig. 1. Besides the data points, the recovered lines using rreaper with the (2,1)-norm (solid line) as data fidelity and the Frobenius norm (dashed line) with parameters $d=1$ and $\alpha = 5$ are presented. In this toy example, rreaper yields nearly a perfect result regardless of the outliers and is in particular more robust than the same model with the Frobenius norm.

### Nuclear Norm and Truncated Hypercube Constraints

**Fig. 2:**
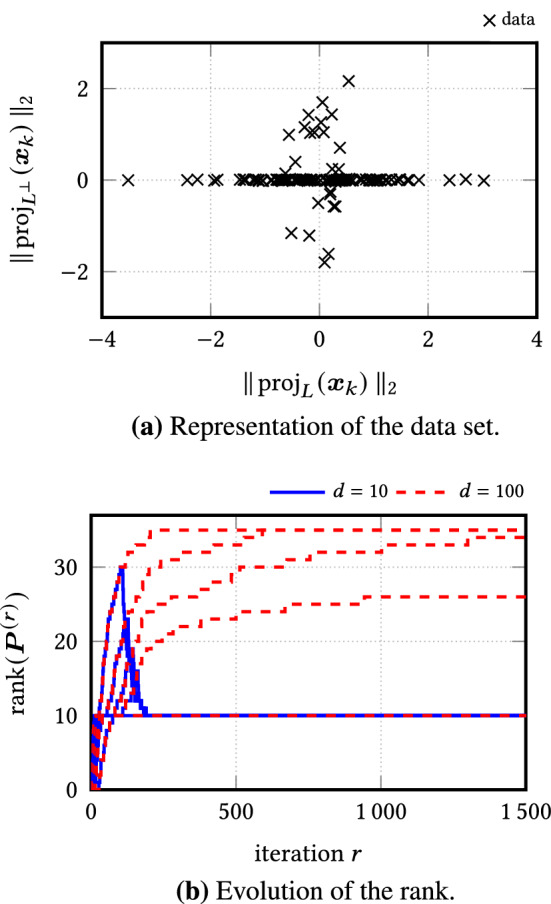
Performance of rreaper for different upper dimension estimators $d=10,100$ and regularization parameters $\alpha = \nicefrac 14, \nicefrac 12, \nicefrac 34 , 1, 2$ (top down for each *d*)

In this example, we are interested how the rank reduction is influenced by the nuclear norm and the projection to the truncated hypercube. In this synthetic experiment, we approximate the given data $\varvec{x}_k \in \mathbb {R}^{100}$ by a 10-dimensional subspace. The data are again generated randomly with respect to the haystack model with $\sigma _{\mathrm {in}} = \sigma _{\mathrm {out}} = 1$ and $\sigma _{\mathrm {noise}} = 0.1$, where 100 points lie near the subspace *L* spanned by the first ten unit vectors and additional 25 outliers. In Fig. 2a, the data set is represented by the distance to the subspace *L* and to the orthogonal complement $L^\perp$.

We apply rreaper in Algorithm 8 with different parameter combination. For the target dimension, we choose in our first experiment $d=10$, which is the wanted dimension, and second one $d=100$, which does not truncate the unit hypercube at all. The influence of the regularization parameter $\alpha$ on the rank of $\varvec{P}^{(r)}$ is shown in Fig. 2b, where the lines from top to down correspond to the regularization parameters $\alpha = \nicefrac 14, \nicefrac 12, \nicefrac 34 , 1, 2$. Since we start the iteration with the zero-rank matrix $\varvec{P}^{(0)} :=\varvec{0}$, the first iterations for $d=10$ and $d=100$ coincides up to the point, where the trace of $\varvec{P}^{(r)}$ exceeds the value 10. The reconstruction errors are recorded in Table 1.

**Table 1:** Reconstruction error $||\hat{\varvec{\varPi }} - \varvec{\varPi }_L||_{{\text {tr}}}$ for different upper dimension estimators $d=10, 100$ and regularization parameters $\alpha = \nicefrac 14, \nicefrac 12, \nicefrac 34 , 1, 2$

dim. *d*	Regularization $\alpha$				
	0.25	0.5	0.75	1	2
10	0.0648	0.0646	0.0645	0.0645	0.0646
100	25.0523	25.0523	24.0569	16.0576	0.0646

Considering only the results for $d=100$ (dashed lines), we see that the nuclear norm reduces the rank of the iteration variable $\varvec{P}^{(r)}$ with an increasing regularization parameter. Further, the rank during the primal-dual algorithm is very sensitive to the regularization parameter. For $d=10$ (solid lines), the situation changes dramatically. After the initial stages, the rank of $\varvec{P}^{(r)}$ decreases nearly to the target dimension. Since the matrices $\varvec{P}^{(r)}$ are no orthogonal projections, rank and trace do not coincide. Due to this fact, the rank is not strictly bounded by the maximal trace of the truncated hypercube. Nevertheless, the projection to the truncated hypercube significantly reduces the rank.

For an optimal rank evolution during the matrix-free primal-dual method, the projection to the truncated hypercube by Algorithm 2 appears to be important. Moreover, the projection makes the rank evolution less sensitive to the regularization parameter $\alpha$ so that a wider range of regularization parameters can be applied without losing the computational benefits of the low rank. Thus, the truncated hypercube projection is an elementary key component of the algorithm.

Finally, we compare the performance of rreaper in this experiment with the other robust PCA methods mentioned above. For this, we repeat the experiment 100 times with random data. For rreaper, we choose the parameters $d=10$ and $\alpha =0.75$. The mean reconstruction errors and the mean run times have been recorded in Table 2. In this experiment, the PCA-L1 fail to approximate the true underlying subspace. Interestingly, rreaper here always yields a slightly better approximation than R1-PCA, FMS, and GGD. The reason for this behavior is that the nuclear norm regularization suppress the Gaussian noise within the computed principle components.

**Table 2:** Mean reconstruction error $||\hat{\varvec{\varPi }} - \varvec{\varPi }_L||_{{\text {tr}}}$ and mean run time (in seconds) for different robust PCA methods

	Mean error	Mean time
rreaper	0.0651	0.5953
PCA-L1	3.4235	0.0490
Nested Weiszfeld	0.3583	0.1613
R1-PCA	0.0662	0.4856
FMS	0.0662	0.0230
GGD	0.0662	0.0149

### Choosing the Parameters

In the last example, we have seen that the target dimension is achieved by choosing either *d* or $\alpha$ appropriately. To understand the relation between the parameters in more detail and finally to get a clue how to choose $\alpha$ if *d* is just taken large enough, we provide some additional experiments.

**Fig. 3:**
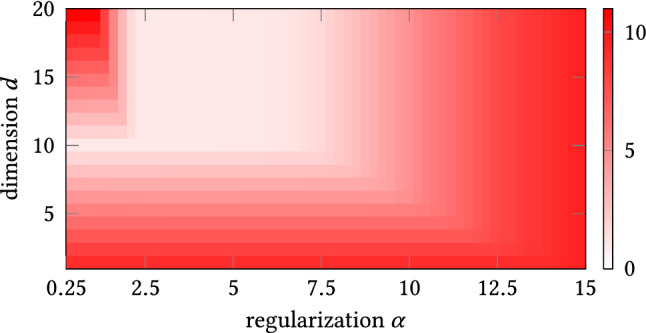
Mean reconstruction error $||\hat{\varvec{\varPi }} - \varvec{\varPi }_L||_{{\text {tr}}}$ for varying $\alpha$ and *d*. For every parameter choice, the experiment has been repeated 25 times

First, we repeat the experiment in Sect. 8.2 sequentially 25 times and consider the mean of the reconstruction error $||\hat{\varvec{\varPi }} - \varvec{\varPi }_L||$ of all 25 experiments, see Fig. 3. For each choice of $\alpha$ and *d*, the experiment has been repeated with the same sets of data. For this specific haystack model, the unknown subspace is recovered by choosing either $d=10$ or $\alpha \in [2.25, 6.75]$. Hence, the first conclusion is that the reconstruction error is much less sensitive to choice of $\alpha$ than to those of *d*.

**Fig. 4:**
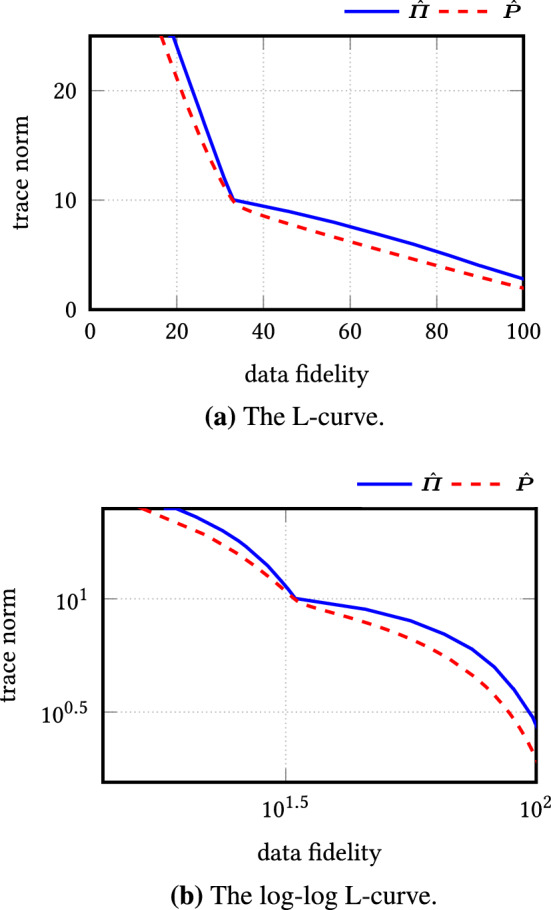
The *L*-curve for $d=25$ of the data set in Sect. 8.2 consisting of 100 inliers ($\sigma _{\mathrm {in}} = 1$) and 25 outliers ($\sigma _{\mathrm {out}} = 1$) with noise $\sigma _{\mathrm {noise}} = 0.1$

**Fig. 5:**
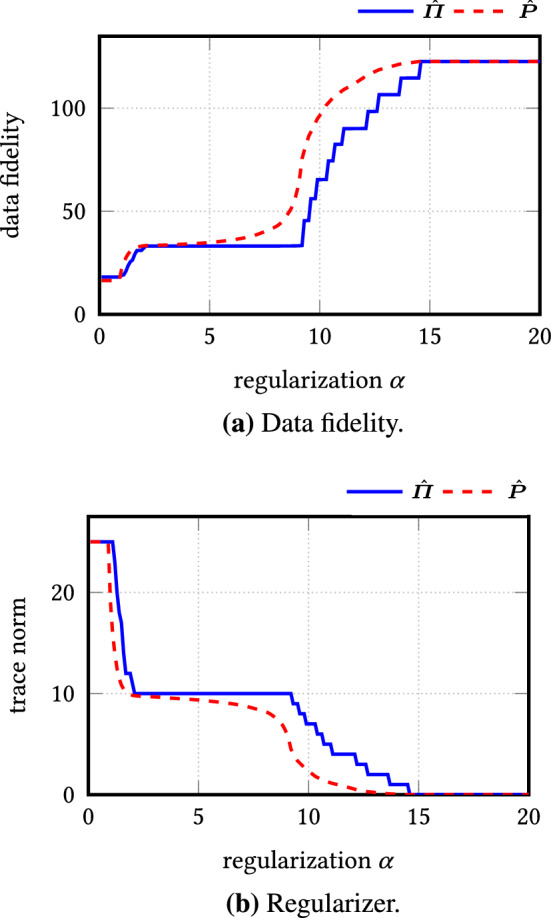
The two components of the *L*-curves in Fig. 4 in dependence of the regularization parameter $\alpha$

Next, considering again the variational nature of rreaper in (9), we recall that $||\varvec{P} \varvec{X} - \varvec{X}||_{2,1}$ describes the error between the ‘projected data’ $\varvec{P} \varvec{X}$ and the given data $\varvec{X}$, and that the $||\varvec{P}||_{{\text {tr}}}$ controls the rank of $\varvec{P}$. In order to find a good subspace, both terms have to be small. Notice that the data fidelity becomes small if the rank/trace becomes large and vice versa. Consequently, $\alpha$ has to be chosen such that both complementary aspects are balanced. One approach coming from inverse problems is the so-called *L*-curve, see for instance [14, 15]. The *L*-curve is a usual or a log-log plot of the data fidelity and the regularizer. For the haystack data set, the corresponding *L*-curve—once computed for the outcome $\hat{\varvec{P}}$ of rreaper, and once for its projection $\hat{\varvec{\varPi }} = {\text {proj}}_{\mathscr {O}_{25}}(\hat{\varvec{P}})$—is shown in Fig. 4, where $d = 25$. Notice that both *L*-curves have a singularity, especially in the log-log plot. For the sake of clarity, we also plotted the data fidelity and the trace norm over $\alpha$, see Fig. 5. The corner of the *L*-curve for $\hat{\varvec{\varPi }}$ corresponds to $\alpha = 9.2$. Further, the *L*-curve slows down at the singularity and is nearly constant for $\alpha \in [2.1,9.2]$. The interval corresponds to the plateaus of the data fidelity and the trace norm in dependence of the regularization parameter $\alpha$, see Fig. 5. At the interval, data fidelity and regularizer are balanced. More importantly, for every $\alpha \in [2.1,9.2]$, the determined subspace has dimension 10 and thus coincides with the true dimension.

We repeated this experiment around 500 times for random chosen *d*, $d_L$, $N_{\mathrm {in}}$, $N_{\mathrm {out}}$, and $\sigma _{\mathrm {noise}}$ and observe the same behavior of the *L*-curve meaning that the edge of the curve has been clearly visible and corresponds to the true subspace dimension. Therefore, it seems that the unknown subspace dimension can be detected and that an appropriate regularization parameter $\alpha$ may be chosen by studying the *L*-curve of the given data set. Notice that the *L*-curve, also well studied in the literature, is a heuristic parameter choice rule. Finally let us mention that if the inlier noise is too large or if the outliers hide the linear structure of the inliers, then the *L*-curve loses its singularity.

### Face Approximation

**Fig. 6:**
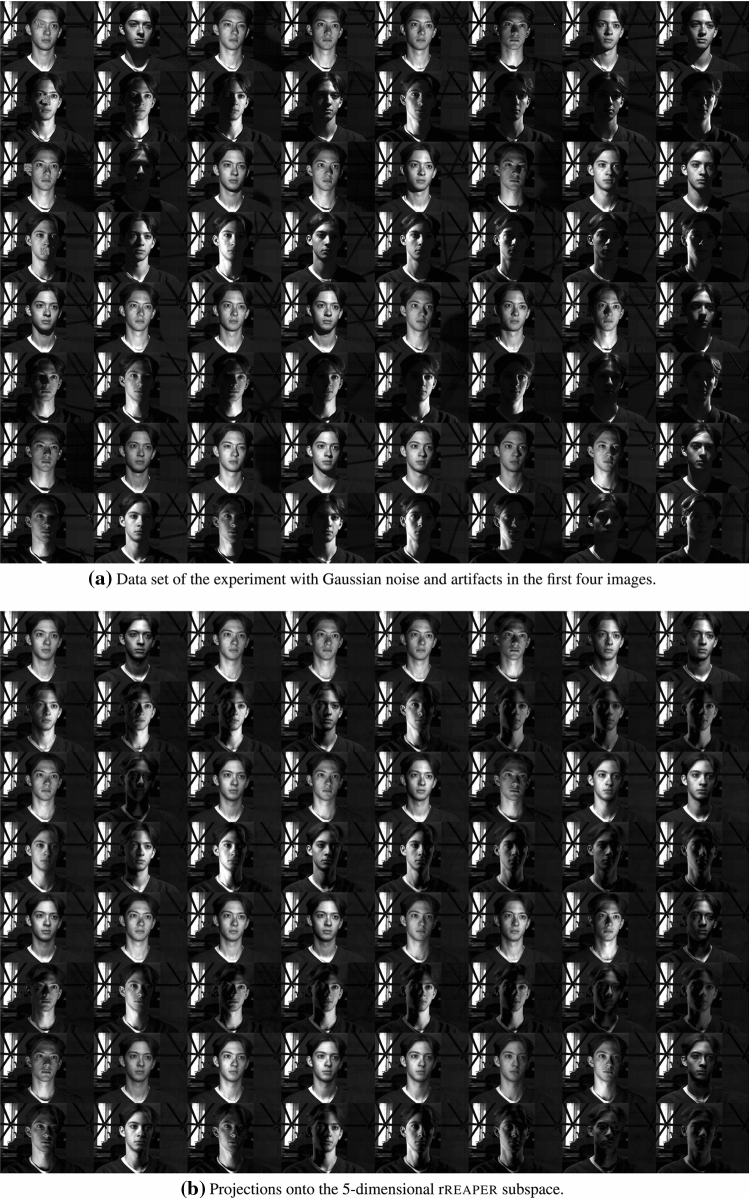
The used data set based on the Extended Yale Face Data Set B and its projections onto the determined subspace using matrix-free rreaper

The idea to use the principle components of face images—the so-called eigenfaces—for recognition, classification and reconstruction was considered in various paper and goes probably back to [45]. In this experiment, we adopt this idea to show that our matrix-free reaper can handle high-dimensional data. Since the computation of an optimal offset is non-trivial as discussed in Sect. 7, we choose just the geometric median. For the experiment, we use the ‘Extended Yale Face Data Set B’ [12, 23] consisting of several series of faces images under different lighting conditions but with the same facial expression. The basis of our noisy data set are 64 images of size $640\times 480$ pixels with integer values between 0 and 255. We place some artifacts in the first four images covering the right eye, the nose, the right ear and the mouth, respectively, and serving as outliers. The complete employed data set is shown in Fig. 6a.

**Fig. 7:**
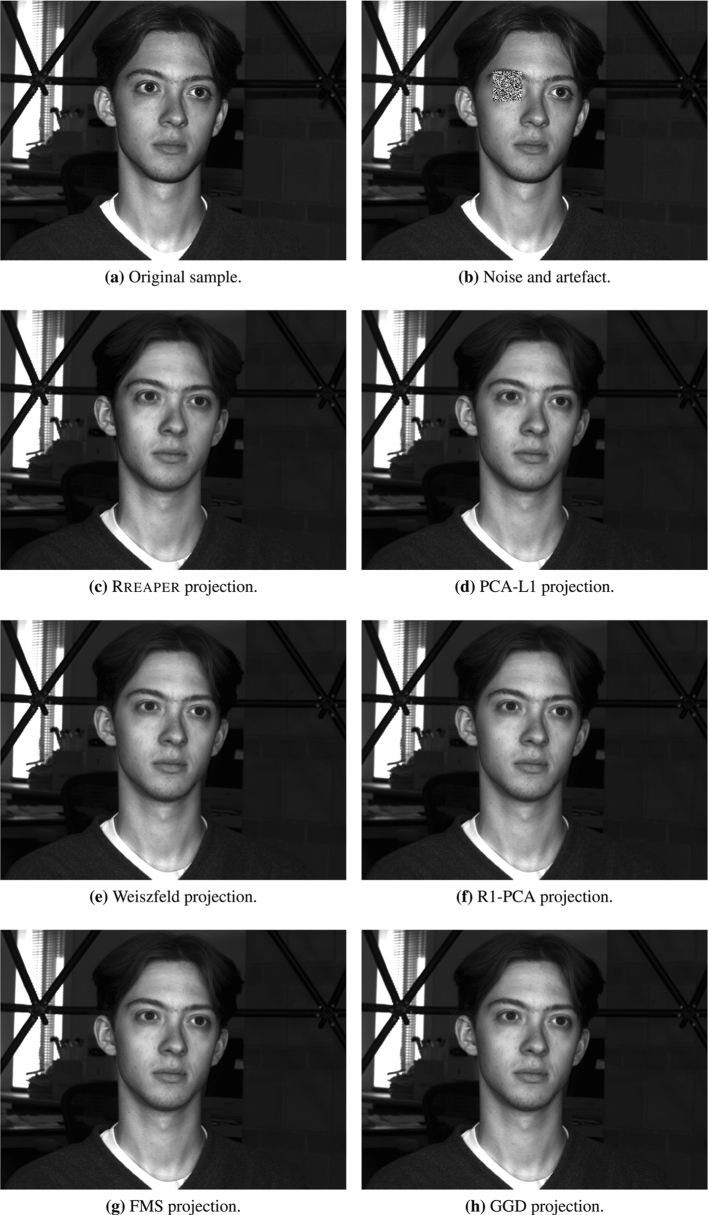
Restoration of the first sample by projecting to the principle components determined by several robust PCA’s

**Fig. 8:**
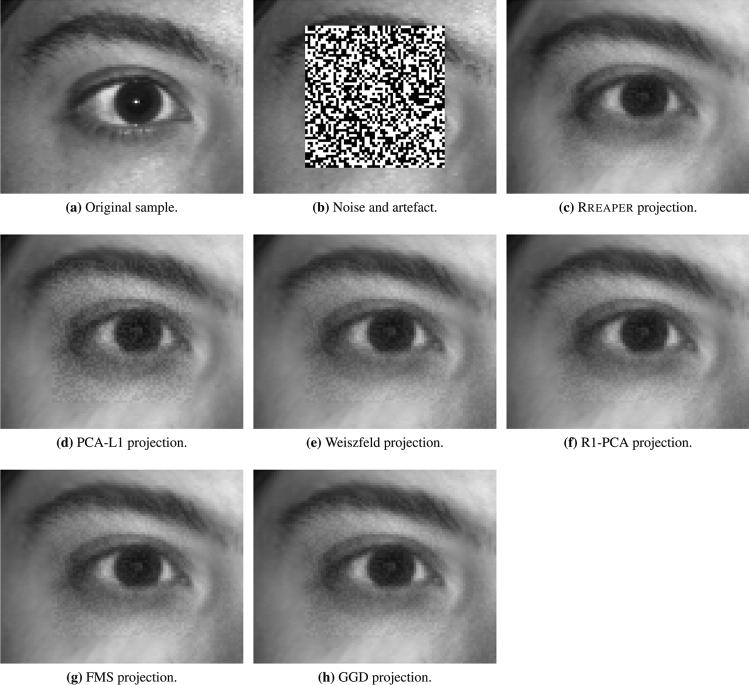
Restoration of the artifact in the first sample by projecting to the principle components determined by several robust PCA’s

It is well-known that such images can be well approximated by a subspace covering around five directions [10]. To remove the artifacts by unsupervised learning, we therefore approximate the full data set including the artificial face images by a five-dimensional subspace ($d=5$) using rreaper and project the corrupted images to the computed subspace. An typical effect of this procedure is that dark regions are lightened, shadows are removed, and reflections at skin and eyes are cleared away. Besides this effects, the major part of the unwanted artefacts is removed, see Fig. 6b. Note that in this example the projection $\hat{\varvec{\varPi }}$ corresponds to a 307,200 $\times$ 307,200 matrix, which would require 703.125 GiB for double precision whereas the matrix-free representation only requires around 14.063 MiB since the rank of the primal variable has been bounded by six due to the choice of $\alpha :=4.2 \cdot 10^4$. The primal-dual algorithm for rreaper has converged after 30 iterations.

Next, we compare rreaper with the robust PCA methods mentioned above. Figure 7 shows examples of the projection of the first corrupted image to the computed subspace of the four considered methods. The restoration of the artifact covering the right eye is shown in Fig. 8 in more details. Taking a closer look in Fig. 8, we notice that PCA-L1, although very fast, yields the worst result, which has also been reported in [35]. The nested Weiszfeld algorithm and R1-PCA nearly remove the artifact. The result of rreaper is comparable but appears to be slightly smoother, which is an effect of the additional regularization.

Comparing the required run times, we notice that PCA-L1 with 10 restarts is the fastest method with 10.189 (in seconds) due to its linear structure, followed by FMS with 12.313, and the nested Weiszfeld algorithm with 18.875. R1-PCA, rreaper and GGD behave similarly with 43.441, 43.439 and 55.968, respectively. Note that the run times are only comparable to a limited extent due to the different nature of the algorithms and hence different stopping criteria. For instance, GGD here requires a very small step size to stay numerically at the Grassmannian. We can clearly see that rreaper is slower than the sequential or heuristic approaches. It seems that the numerical effort of rreaper is however comparable with the non-convex methods like R1-PCA. Rreaper converges however to a global minimizer, where the non-convex methods may be stuck in local minima, which here happens for GGD.

## Conclusion

Convex models are usually preferable over non-convex ones due to their unique local minimum. While robust PCA models that can handle high-dimensional data are usually non-convex, a convex relaxation was proposed by the reaper model. Relying on the projector approach, it is however not applicable for high-dimensional data in its original form. To manage such data, we have combined primal-dual optimization techniques from convex analysis with sparse factorization techniques from the Lanczos algorithm. Moreover, we extended the model by penalizing the nuclear norm of the operator and called it rreaper. This results in the first convex variational method for robust PCA that can handle high-dimensional data since the required memory is reduced from $O(nN + n^2)$ to $O(nN + nr)$, where *r* is the maximal rank during the primal-dual iteration and has usually the same magnitude as *d*. The rreaper minimization algorithm always converges to a global minimizer of the regularized objective. Moreover, using the *L*-curve method an advantage of the new method seems to be that the dimension of the low-dimensional subspace must not be known in advance, but may be overestimated. We intend to further investigate this direction from the point of view of multi-objective optimization [8, 48]. We addressed the problem of the bias in robust PCA, but more research in this directions appears to be necessary. Further other sparsity promoting norms then the nuclear norm could be involved. Our method can be enlarged to 3D images as videos, 3D stacks of medical or material images, where tensor-free methods will come into the play. Finally, it may be interesting to couple PCA ideas with approaches from deep learning to better understand the structure of both.
